# Comparative analysis of TTF‐1 binding DNA regions in small‐cell lung cancer and non‐small‐cell lung cancer

**DOI:** 10.1002/1878-0261.12608

**Published:** 2019-12-15

**Authors:** Satoshi Hokari, Yusuke Tamura, Atsushi Kaneda, Akihiro Katsura, Masato Morikawa, Fumihiko Murai, Shogo Ehata, Shuichi Tsutsumi, Yuichi Ishikawa, Hiroyuki Aburatani, Toshiaki Kikuchi, Kohei Miyazono, Daizo Koinuma

**Affiliations:** ^1^ Department of Molecular Pathology Graduate School of Medicine The University of Tokyo Japan; ^2^ Department of Respiratory Medicine and Infectious Diseases Niigata University Graduate School of Medical and Dental Sciences Japan; ^3^ Department of Molecular Oncology Graduate School of Medicine Chiba University Japan; ^4^ Genome Science Division Research Center for Advanced Science and Technology The University of Tokyo Japan; ^5^ Division of Pathology the Cancer Institute Hospital Japanese Foundation for Cancer Research Tokyo Japan

**Keywords:** ASCL1, ChIP‐seq, lung cancer, NKX2‐1, SCLC, TTF‐1

## Abstract

Thyroid transcription factor‐1 (TTF‐1, encoded by the *NKX2‐1* gene) is highly expressed in small‐cell lung carcinoma (SCLC) and lung adenocarcinoma (LADC), but how its functional roles differ between SCLC and LADC remains to be elucidated. Here, we compared the genome‐wide distributions of TTF‐1 binding regions and the transcriptional programs regulated by TTF‐1 between NCI‐H209 (H209), a human SCLC cell line, and NCI‐H441 (H441), a human LADC cell line, using chromatin immunoprecipitation‐sequencing (ChIP‐seq) and RNA‐sequencing (RNA‐seq). TTF‐1 binding regions in H209 and H441 cells differed by 75.0% and E‐box motifs were highly enriched exclusively in the TTF‐1 binding regions of H209 cells. Transcriptome profiling revealed that TTF‐1 is involved in neuroendocrine differentiation in H209 cells. We report that TTF‐1 and achaete‐scute homolog 1 (ASCL1, also known as ASH1, an E‐box binding basic helix–loop–helix transcription factor, and a lineage‐survival oncogene of SCLC) are coexpressed and bound to adjacent sites on target genes expressed in SCLC, and cooperatively regulate transcription. Furthermore, TTF‐1 regulated expression of the Bcl‐2 gene family and showed antiapoptotic function in SCLC. Our findings suggest that TTF‐1 promotes SCLC growth and contributes to neuroendocrine and antiapoptotic gene expression by partly coordinating with ASCL1.

## Graphical abstract

A remarkable difference in the thyroid transcription factor‐1 (TTF‐1) binding regions on the genome was identified between lung adenocarcinoma (LDAC) and small‐cell lung carcinoma (SCLC). TTF‐1 inhibits epithelial‐mesenchymal transition in LADC cells, while TTF‐1 cooperates with ASCL1 to induce neuroendocrine differentiation and antiapoptotic function in SCLC cells.

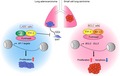

**Abbreviations**
ASCL1: achaete‐scute homolog 1
CCLE: cancer cell line encyclopedia
ChIP‐seq: chromatin immunoprecipitation‐sequencing
EMT: epithelial–mesenchymal transition
GO: gene ontology
IB: immunoblotting
IHC: immunohistochemistry
IP: immunoprecipitation
LADC: lung adenocarcinoma
NSCLC: non‐small‐cell lung cancer
PLA: proximity ligation assay
qRT‐PCR: quantitative real‐time reverse transcription–PCR
RNA‐seq: RNA‐sequencing
SCLC: small‐cell lung cancer
TF: transcription factor
TGF‐β: transforming growth factor‐β
TRU: terminal respiratory unit
TTF‐1: thyroid transcription factor‐1

## 1 Introduction

Thyroid transcription factor‐1 (TTF‐1, encoded by the *NKX2‐1* gene) is a homeodomain‐containing master transcription factor (TF) of lung morphogenesis and differentiation of pulmonary epithelial cells (Kimura *et al.*, 1996; Minoo *et al.*, 1999). TTF‐1 is expressed in 75–80% of lung adenocarcinoma (LADC), a non‐small‐cell lung cancer (NSCLC) subtype, and is a marker of the terminal respiratory unit (TRU) subtype (Yatabe *et al.*, 2002). TTF‐1‐positive LADC patients show better prognosis than TTF‐1‐negative LADC patients (Kim *et al.*, 2018; Zhang *et al.*, 2015). TTF‐1 reduces invasion and metastasis in LADC (Hosono *et al.*, 2012; Winslow *et al.*, 2011); TTF‐1 inhibits TGF‐β‐induced epithelial–mesenchymal transition (EMT) in LADC cells (Isogaya *et al.*, 2014; Saito *et al.*, 2009). In contrast, the *NKX2‐1* gene is amplified in 10–15% of LADCs and acts as a lineage‐survival oncogene (Kwei *et al.*, 2008; Tanaka *et al.*, 2007). TTF‐1 has a prosurvival function in cancer cells via *ROR1* induction and *LMO3* oncogene regulation (Watanabe *et al.*, 2013; Yamaguchi *et al.*, 2012). Thus, TTF‐1 plays a double‐edged role in LADC (Yamaguchi *et al.*, 2013).

Although small‐cell lung cancer (SCLC) has primitive neuroendocrine features distinct from LADC, 80–90% of SCLC tumors express pathologically high levels of TTF‐1 (Misch *et al.*, 2015). TTF‐1 is expressed not only in SCLC but also in small‐cell carcinoma of other organs, such as prostate (Wang and Epstein, 2008). Similar to achaete‐scute homolog 1 (ASCL1, also known as ASH1), the NKX‐homeodomain family TFs play a critical role in reprogramming normal human epithelial tissues to a neuroendocrine cancer lineage (Park *et al.*, 2018), suggesting a critical function of TTF‐1 in SCLC other than promoting epithelial cell differentiation. Conversely, the majority of SCLCs are of the peripheral type, and the peripheral‐type SCLC expresses TTF‐1 more frequently than does the central‐type (Miyauchi *et al.*, 2015), indicating that most SCLCs are derived from TRU cells expressing TTF‐1. A recent report revealed that the expression of TTF‐1 is positively regulated by ASCL1 in SCLC cell lines to induce nuclear factor I B‐type (NFIB) (Horie *et al.*, 2018).

The aforementioned studies strongly suggest a central role of TTF‐1 in SCLC pathology. However, the difference in the roles of TTF‐1 between SCLC and LADC remains to be elucidated. Employing chromatin immunoprecipitation‐sequencing (ChIP‐seq) and RNA‐sequencing (RNA‐seq), here we compared the genome‐wide TTF‐1 binding profiles and the TTF‐1‐mediated transcriptional programs in SCLC and LADC cell lines.

## 2 Materials and methods

### 2.1 Cell culture

Human SCLC NCI‐H209 (H209) and NCI‐H345 (H345), and NSCLC A549 and NCI‐H441 (H441) were obtained from American Type Culture Collection (Manassas, VA, USA). Human SCLC Lu‐135 and STC‐1 cells were purchased from the Japanese Collection of Research Bioresources (JCRB) Cell Bank (Osaka, Japan). H209, H441, Lu‐135, and STC‐1 cells were cultured in RPMI 1640 (#11875; Thermo Fisher Scientific, Waltham, MA, USA). H345 cells were cultured in Dulbecco’s modified Eagle’s medium (DMEM)/Nutrient Mixture F‐12 (1 : 1) medium (#11330; Thermo Fisher Scientific) with 5 μg·mL^−1^ insulin, 5 μg·mL^−1^ transferrin, 30 nm sodium selenite (#I1884; Sigma‐Aldrich, St. Louis, MO, USA), 10 nm β‐estradiol (#E2257; Sigma‐Aldrich), and 10 nm hydrocortisone (#H0135; Sigma‐Aldrich). A549 cells were cultured in DMEM (#11965; Thermo Fisher Scientific). All culture media included 10% FBS (#SH30910.03; Thermo Fisher Scientific), 100 U·mL^−1^ penicillin G, and 100 µg·mL^−1^ streptomycin. All cells were maintained in a humidified atmosphere of 5% CO_2_ at 37 °C.

### 2.2 Clinical samples

This study was certified by Ethics Committee in the University of Tokyo and in the Cancer Institute and was carried out in accordance with the Helsinki Declaration. Primary SCLC samples were obtained from patients undergoing pulmonary resection at the Department of Surgery, Cancer Institute Hospital, with written informed consent. These samples were immediately frozen with liquid nitrogen and stored at −80 °C. The frozen materials were microscopically examined by two independent pathologists and were dissected to enrich cancer cells when necessary. Total RNA was extracted using TRIzol (Thermo Fisher Scientific), confirming high quality of RNA with RNA intensity number ≥ 7.0, and expression array analysis using Affymetrix GeneChip Human Genome U133 Plus 2.0 oligonucleotide arrays (Fremont, CA, USA) was conducted previously (Sato *et al.*, 2013). In overall survival analysis, the Kaplan–Meier curve was drawn and *P*‐value was calculated by log‐rank test using r (version 2.15.3) with ‘survival’ package (version 2.41.3) (http://www.R-project.org/). The end of follow‐up period was 142 months from the primary surgery, and the mean follow‐up time of the cases was 92 months. Death as a result of SCLC was the primary end point, and deaths by other causes were censored.

### 2.3 Antibodies

The following antibodies were used: anti‐TTF‐1 [for immunoblotting (IB) and ChIP, 1 : 1000 and 5 µg, respectively, #MS‐699‐P; Lab Vision Corporation, Fremont, CA, USA; for immunohistochemistry (IHC) (1 : 50), immunofluorescence (1 : 200), and *in situ* proximity ligation assay (PLA) (1 : 100), #ab76013; Abcam, Cambridge, UK], anti‐α‐tubulin (1 : 10 000, #T1699; Sigma‐Aldrich), anti‐FLAG M2 (1 : 1000, #F3165; Sigma‐Aldrich), anti‐c‐Myc (1 : 1000, #017‐21874; Wako Pure Chemical Industries, Osaka, Japan), anti‐MASH1/ASCL1 [for PLA (1 : 50), IB (1 : 1000), and ChIP (5 µg), #556604; BD, Franklin Lakes, NJ, USA], anti‐Bim (1 : 1000, #2933; Cell Signaling Technology, Danvers, MA, USA), and anti‐Bcl‐2 (1 : 100 for IHC, 1 : 1000 for IB, and 1 : 400 for immunofluorescence, #15071; Cell Signaling Technology).

### 2.4 Immunohistochemistry of tissue microarray

A tissue microarray of SCLC (LC818a) was obtained from US Biomax (Rockville, MD, USA). The array was deparaffinized and rehydrated followed by antigen retrieval using 10 mm sodium citrate buffer (pH 6.0). Endogenous peroxidase activity was blocked by 3.0% hydrogen peroxide. The array was then blocked with Blocking One reagent (Nacalai Tesque, Kyoto, Japan) and incubated with anti‐TTF‐1, anti‐MASH1/ASCL1, or anti‐Bcl‐2 antibody. Vectastain ABC Kit (Vector Laboratories Inc., Burlingame, CA, USA) and 3,3′‐diaminobenzidine (Dako, Agilent Technologies, Santa Clara, CA, USA) were used for immunodetection. Sections were weakly counterstained with hematoxylin. Images were captured with the all‐in‐one fluorescence microscope, BZ‐X710 (Keyence, Osaka, Japan).

We evaluated three spots per tumor sample with a 20× objective. For TTF‐1 and ASCL1 IHC, the fraction of stained tumor cells was scored as follows: 0, 0%; 1, 1–20%; 2, 21–50%; 3, 51–80%; and 4, > 81%. For Bcl‐2 IHC, the intensity of staining was scored as follows: 0, negative; 1, weak; 2, moderate; 3, strong; and 4, very strong. The IHC scores of each array spot were evaluated by a pulmonologist (S.H.).

### 2.5 Immunofluorescence

Paraffin‐embedded H209 cells were treated as described above. The cells were stained with anti‐TTF‐1 and anti‐Bcl‐2 antibodies. Stained cells were visualized using anti‐mouse IgG H&L (Alexa Fluor 594; Thermo Fisher Scientific), anti‐rabbit IgG H&L (Alexa Fluor 488; Thermo Fisher Scientific), and DAPI. Images were captured with the all‐in‐one fluorescence microscope BZ‐X710. The expression of Bcl‐2 was quantified by area fraction measurement of ImageJ and normalized by cell number. For each condition, randomly selected two enlarged images were used for calculation.

### 2.6 In situ proximity ligation assay

We used Duolink kit (Olink, Uppsala, Sweden) for in situ PLA assay as previously described (Isogaya *et al.*, 2014). The anti‐TTF1 and anti‐MASH1/ASCL1 were used as primary antibodies. Combination of the primary antibodies was determined so that no antibody cross‐reacted with the PLA probe‐conjugated secondary antibody to other primary antibodies. Vectashield mounting medium with DAPI (Vector Laboratories) was used as a nuclear counterstain. The experiment was performed twice with essentially the similar results.

### 2.7 Immunoblotting and immunoprecipitation

For IB, cells were rinsed with ice‐cold PBS and lysed with RIPA buffer [50 mm Tris/HCl (pH 8.0), 150 mm NaCl, 1% Nonidet P‐40, 0.1% SDS, and 0.5% sodium deoxycholate] that included cOmplete EDTA‐free protease inhibitor (Roche Diagnostics, Basel, Switzerland). After centrifugation at 15 000 r.p.m. (20 400 ***g***) and 4 °C for 10 min, protein concentrations were estimated using the BCA Protein Assay Kit (Thermo Fisher Scientific). The same amount of proteins was subjected to SDS/polyacrylamide gel electrophoresis and transferred to Fluoro Trans W membranes (Pall, Port Washington, NY, USA). For immunoprecipitation (IP), cultured cells were lysed with lysis buffer [1% Nonidet P‐40, 150 mm NaCl, 20 mm Tris/HCl (pH 7.5), and cOmplete EDTA‐free protease inhibitor]. Co‐IP was performed as previously described (Koinuma *et al.*, 2009a). IB was carried out as described previously (Katsura *et al.*, 2017; Koinuma *et al.*, 2011) and imaged with a LAS‐4000 lumino Image analyzer (FUJIFILM, Tokyo, Japan). The experiments were repeated, and the representative data are shown in the figures.

### 2.8 RNA interference

Reverse transfection of Stealth Select siRNA (Thermo Fisher Scientific) was performed using Lipofectamine RNAiMAX (Thermo Fisher Scientific). We used two sets of siRNA: TTF‐1 (siTTF‐1) (#1: HSS144277 and #2: HSS144278) and ASCL1 (siASCL1) (#1: HSS100745 and #2: HSS181121). Medium GC Complex #2: 12935‐112 (Thermo Fisher Scientific) was used as negative control siRNA (siNC).

### 2.9 RNA extraction and quantitative real‐time reverse transcription–PCR

Total RNA was extracted with the RNeasy Mini Kit (Qiagen, Hilden, Germany). First‐strand cDNAs were synthesized using PrimeScript II reverse transcriptase and oligo dT primers (Takara Bio, Shiga, Japan) according to the manufacturer’s instructions. Quantitative real‐time reverse transcription–PCR (qRT‐PCR) was performed with the StepOnePlus Real‐Time PCR System (Thermo Fisher Scientific) and the FastStart Universal SYBR Green Master Mix (ROX) (Roche Diagnostics). All samples were run in duplicate, and results were averaged and normalized to the expression of *GAPDH* (glyceraldehyde‐3‐phosphate dehydrogenase). Primer sequences are shown in Table S1.

### 2.10 Chromatin immunoprecipitation, ChIP‐seq, and data analysis

ChIP‐qPCR and ChIP‐seq of H441 and H209 cells were performed using anti‐TTF‐1 antibody or anti‐ASCL1 antibody as described previously (Koinuma *et al.*, 2009b). Data were obtained as two biological replicates. The TTF‐1 ChIP‐seq of H441 cells has been published [available at Gene Expression Omnibus (GEO; http://www.ncbi.nlm.nih.gov/geo/query/acc.cgi?acc=GSE51510)] (Isogaya *et al.*, 2014), and we additionally obtained new data as a biological replicate in this study. For ChIP‐seq data analysis, reads were trimmed down to 50 bp to compare with the published datasets and were aligned against the human reference genome (NCBI Build 36, hg19) with bowtie (Langmead *et al.*, 2009). Peaks of TTF‐1 and ASCL1 ChIP‐seq were called using MACS2 (Zhang *et al.*, 2008) by two‐sample analysis using default parameters, where input genomic DNA was used as a negative control. Mapped sequence data were visualized using Integrative Genomics Browser. Gene annotations and gene ontology (GO) analysis for TTF‐1 and ASCL1 ChIP‐seq data were performed using great version 3.0.0 with default parameters and whole genome as background (McLean *et al.*, 2010). *De novo* motif discovery and motif centrality analysis for TTF‐1 and ASCL1 ChIP‐seq were conducted with meme‐chip ver 5.0.5 (Machanick and Bailey, 2011), which internally used dreme version 5.0.5 and centrimo version 5.0.5 (Bailey and Machanick, 2012). The 500‐bp genomic sequences flanking the peak summits of the binding regions were used for calculation. Default parameters were used except for the number of motifs (8) and the minimal length of the motif (5) for meme. Primer sequences for ChIP‐qPCR are shown in Table S2. The full list of the motifs reported by dreme is available as Data S1–S3.

### 2.11 RNA‐seq and data analysis

RNA‐seq was performed as described previously (Isogaya *et al.*, 2014; Kawasaki *et al.*, 2018). For RNA‐seq data analysis, reads were aligned against the human genome (NCBI Build 36, hg19) using tophat2 (https://ccb.jhu.edu/software/tophat/). Differential expression was evaluated using the Cuffdiff function of cufflinks (http://cufflinks.cbcb.umd.edu/). Gene set enrichment analysis (GSEA) (Subramanian *et al.*, 2005) was used for gene functional classification.

### 2.12 Plasmid construction and cDNA transfection

Plasmids encoding human TTF‐1 and ASCL1 were constructed by PCR amplification. Fragments were subcloned into pcDNA3‐6xMyc vector (TTF‐1) or pcDNA3‐FLAG vector (ASCL1). All cDNAs were verified by sequencing. Transient transfection into cells was performed using Lipofectamine 3000 reagent (Thermo Fisher Scientific), as recommended by the manufacturer’s protocol. Plasmids for adenoviral expression vectors of LacZ and TTF‐1 were prepared as previously described (Saito *et al.*, 2009).

### 2.13 Adenovirus production and infection

Adenoviruses for transduction of LacZ (Ad‐LacZ) or TTF‐1 (Ad‐TTF‐1) were generated using ViraPower Adenoviral Expression System (Thermo Fisher Scientific). Adenovirus titer was determined using the Adeno‐X Rapid Titer Kit (Takara‐Clontech, Shiga, Japan). Multiplicity of infection 200 was used for infection using Ad‐TTF‐1. The same corresponding multiplicity of infection for Ad‐LacZ was used as control.

### 2.14 Cell proliferation assay

H209 cells were seeded at a density of 1 × 10^5^ per well into a 24‐well plate with siRNA transfection. After 72 h, cell proliferation was evaluated with Cell Count Reagent SF (Nacalai Tesque). Absorbance at 450 nm was measured with a Model 680 Microplate Reader (Bio‐Rad, Melville, NY, USA), and the absorbance at 595 nm was deducted from it.

### 2.15 Apoptosis assay

H209 cells were seeded at a density of 5 × 10^5^ per well into a 6‐well plate with siRNA transfection. After 72 h, cells were stained with Annexin V and propidium iodide (eBioscience, Vienna, Austria) for 15 min at room temperature prior to detection in Gallios (Beckman Coulter, Brea, CA, USA). Results were analyzed using the flowjo software (BD).

### 2.16 Statistical analysis

Student’s *t*‐test was used for two‐sample analyses. Comparisons of the multiple experimental groups were made using one‐way ANOVA with Dunnett’s test. The Mann–Whitney *U*‐test was used for IHC score data. Comparisons of the frequency were made using the chi‐square test. Statistical analyses were conducted with prism 7.00 (GraphPad Software, San Diego, CA, USA).

## 3 Results

### 3.1 TTF‐1 is highly expressed in a subset of SCLC in association with poor prognosis

**Figure 1:**
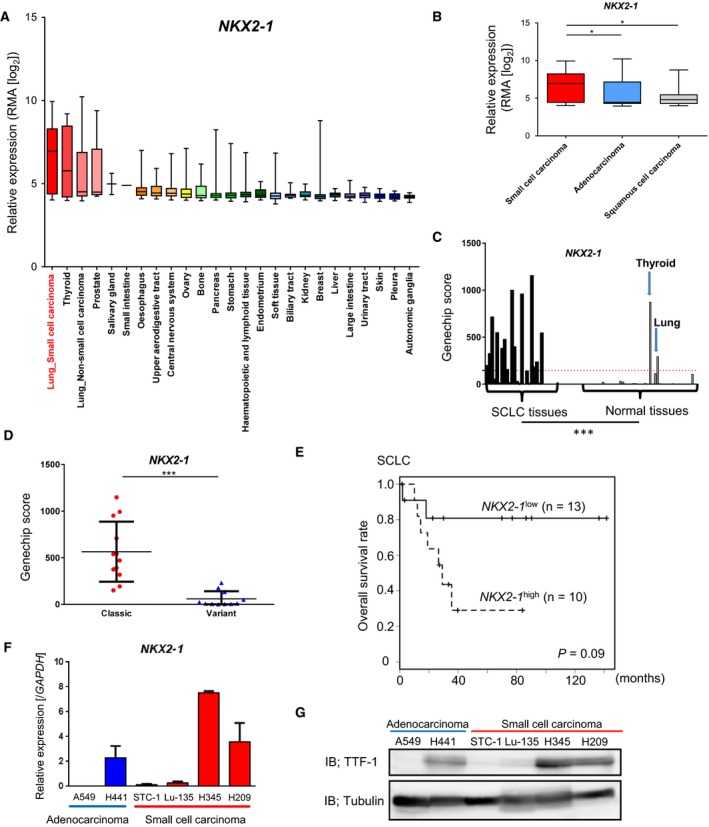
High expression of *NKX2‐1* in a subset of SCLC. (A) Expression of *NKX2‐1* mRNA (encoding TTF‐1) in various cancers from the CCLE database. Normalized expression of the microarray data was calculated by robust multichip analysis (RMA). (B) Lung cancer cell datasets from CCLE were divided into SCLC (*n* = 52), LADC (*n* = 73), and squamous cell carcinoma (*n* = 28). *P < 0.05, one‐way ANOVA with Dunnett’s test. (C) Expression of *NKX2‐1* in 23 clinical SCLC tumors and 42 normal tissues (http://www.ncbi.nlm.nih.gov/geo/query/acc.cgi?acc=GSE43346). Red dotted bar indicates the average expression of *NKX2‐1* in the normal tissues. ***P < 0.001, unpaired *t*‐test. (D) Comparison of *NKX2‐1* expression in the clinical SCLC samples (http://www.ncbi.nlm.nih.gov/geo/query/acc.cgi?acc=GSE43346) between the classic (*n* = 12) and variant (*n* = 11) subtypes. Bars indicate the mean and S.E. ***P < 0.001, unpaired *t*‐test. (E) Relationship between *NKX2‐1* expression and overall survival in SCLC patients (http://www.ncbi.nlm.nih.gov/geo/query/acc.cgi?acc=GSE43346) (Sato et al., 2013) analyzed by the Kaplan–Meier plot. Patients were divided into *NKX2‐1* ^low^ (GeneChip score < 250, *n* = 13) and *NKX2‐1* ^high^ (score > 250, *n* = 10). *P*‐value was calculated by log‐rank test. (F) qRT‐PCR analysis of *NKX2‐1* mRNA in lung cancer cells used in this study. Data represent means of the two biological replicates. Error bars, SE. (G) IB for TTF‐1 in the lung cancer cell lines.

Investigation of microarray data from the Cancer Cell Line Encyclopedia (CCLE) (Barretina *et al.*, 2012) showed that the median values of *NKX2‐1* expression in lung cancers were higher among cancers originating from different tissues (Fig. 1A), and SCLC showed significantly higher expression than other types of lung cancers (Fig. 1B). The expression of *NKX2‐1* in clinical SCLC tumors was also higher than that in normal tissues except in thyroid and lung, which physiologically express TTF‐1 (Fig. 1C) (Sato *et al.*, 2013). Our previous microarray dataset of SCLC tumors revealed that the classic‐type SCLC was characterized with lower *AJUBA* (also known as *JUB*) and higher *GRP* expression when compared with the variant SCLC group. Classic‐type SCLC was associated with poor prognosis, and *NKX2‐1* expression was significantly higher in this type (Fig. 1D) (Sato *et al.*, 2013). Although a recently published paper reported the association of high *NKX2‐1* expression and poor SCLC patient prognosis (Yan *et al*, 2019), *NKX2‐1* expression was not significantly associated with overall survival in this cohort (Fig. 1E) (log‐rank test, *P* = 0.09).

We then investigated the expression of *NKX2‐1* in LADC and SCLC cell lines. H209 and H345 cells (classified as classic‐type SCLC cell lines according to the neuroendocrine feature) (Horie *et al.*, 2016) highly expressed *TTF‐1* mRNA and protein (Fig. 1F,G).

### 3.2 TTF‐1 binding regions in LADC and SCLC cells show little overlap in distribution

**Figure 2:**
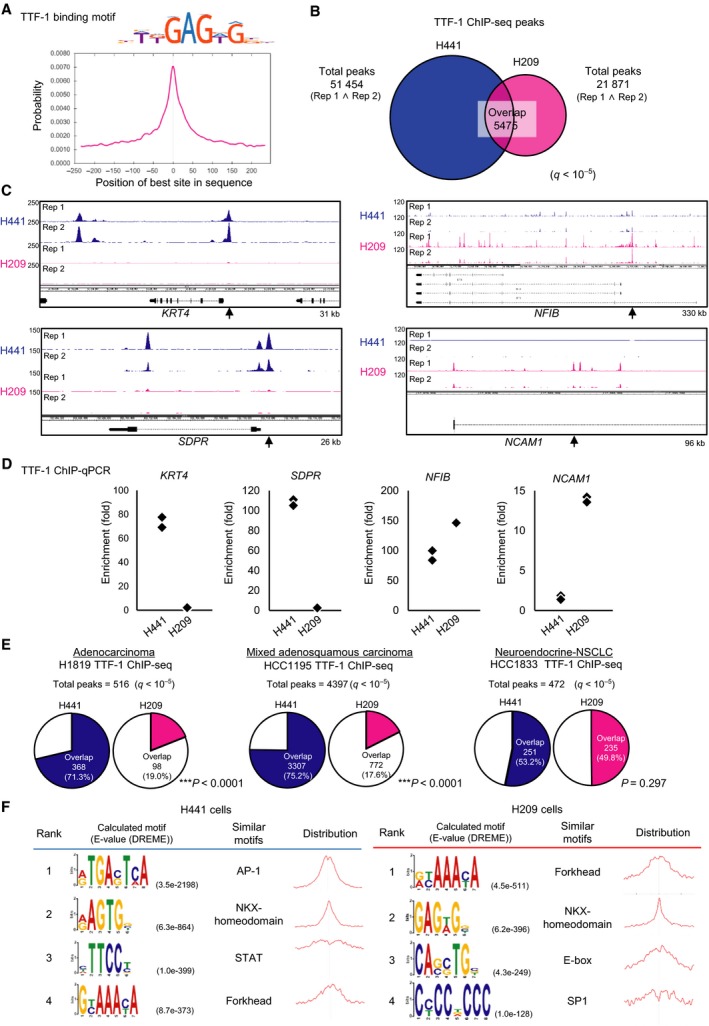
Distinct properties of TTF‐1 binding regions in SCLC. (A) Motif centrality analysis of TTF‐1 binding regions using CentriMo. The known TTF‐1 binding motif from HOCOMOCOv11 (NKX21_HUMAN.H11MO.0.A, upper panel) was used for calculation. The *x*‐axis indicates the relative position (bp) of the best site from the peak summit of each binding region. (B) Venn diagram showing the overlap of TTF‐1 ChIP‐seq peaks between H209 and H441. (C) The upper or lower two lanes show TTF‐1 binding signals of the two biological replicates (Rep 1 and Rep 2) obtained from H441 (blue) or H209 (magenta) cells, respectively. Arrows show the position of ChIP‐qPCR analysis evaluated in (D). kb Sizes denote the ranges shown in the panels. (D) ChIP‐qPCR analysis of TTF‐1 binding in H441 and H209 cells. Data represent the result of two biological replicates. %input values at the target genomic loci were normalized to those at *HBB* locus. (E) Charts showing the proportion of overlaps with TTF‐1 binding regions in NSCLC cell lines. The binding regions of TTF‐1 were identified by ChIP‐seq data using H441 and H209 cells (this study) or other NSCLC cell lines (SRP045118). The proportions of overlaps with the TTF‐1 binding regions in H441 and H209 cells were compared. ***P < 0.0001, chi‐square test. (F) Motif enrichment and centrality analysis using DREME and CentriMo. The top four *de novo*‐calculated motifs with the smallest *E*‐values identified by DREME are shown.

Genome‐wide distribution of TTF‐1 binding regions in SCLC and LADC cells was compared by ChIP‐seq in the H209 and H441 cell lines. Binding regions of 48 421 and 26 752 were identified from each of the two biological replicates in H209 cells (*q* < 10^−5^). In H441 cells, 58 099 and 74 258 binding regions were identified in each of the two biological replicates (*q* < 10^−5^). We calculated the intersection of the biological replicates for each cell line and used 21 871 (H209) and 51 454 (H441) TTF‐1 binding regions in the following analyses, respectively. The known NKX2‐1 motif was enriched and had a centrality for both cell lines (Fig. 2A), supporting the validity of the data. Significant peaks were found at known TTF‐1 binding sites in H441 LADC cells, for example, the *SFTPB* gene (encoding surfactant protein B) locus but not at the *HBB* gene locus used as a negative control (Fig. S1A). In contrast, TTF‐1 binding at *SFTPB* locus in H209 cells was not significant at the present settings (Fig. S1A) and ChIP‐qPCR suggested very weak binding compared to H441 cells (Fig. S1B).

Surprisingly, comparisons between H441 and H209 cells revealed little overlap of the TTF‐1 binding regions at the genome‐wide level (Fig. 2B). *KRT4* and *SDPR* gene loci, which were TTF‐1‐bound genes in LADC cells (Isogaya *et al.*, 2014), had peaks only in H441 cells, whereas *NFIB* and *NCAM1* gene loci, which are known as oncogenes for SCLC (Calbo *et al.*, 2011; Semenova *et al.*, 2016), had peaks mainly in H209 cells (Fig. 2C). We confirmed the similar tendency by ChIP‐qPCR (Fig. 2D). Furthermore, comparisons using published TTF‐1 ChIP‐seq data of other NSCLC cell lines (SRP045118) (Clarke *et al.*, 2015) revealed that while TTF‐1 binding regions in NCI‐H1819 and HCC1195 cells showed more overlap with H441 cells, TTF‐1 binding regions in HCC1833 cells, which were derived from LADC with neuroendocrine features (Kosari *et al.*, 2014), showed comparable ratios of overlap in H209 and H441 cells (Fig. 2E). These findings suggest that TTF‐1 plays highly different roles in LADC and SCLC.

*De novo* motif analysis of the TTF‐1 binding regions in H441 and H209 cells was performed to understand the differences in their binding sequence preferences. Predictably, NKX‐homeodomain motif emerged as one of the top preferred motifs in both cell lines, while the Forkhead family TFs (the known cooperative TFs of TTF‐1) binding motifs were also commonly identified (Fig. 2F). Among differentially preferred motifs, AP‐1 was strongly enriched in H441 cells, consistent with a previous report (Maeda *et al.*, 2012), whereas the E‐box motif was enriched specifically in H209 cells. Both ASCL1 and NEUROD1, members of the E‐box binding TFs, were the lineage‐specific TFs and differentially regulate key oncogenes in SCLC (Borromeo *et al.*, 2016), suggesting that TTF‐1 plays a role in the development of SCLC through these TFs.

Gene ontology analysis of nearby genes calculated from the ChIP‐seq data indicated that in H209 cells, the TTF‐1‐bound genes were related to the biological process terms related to neuron differentiation and aorta morphogenesis (Fig. S1C), suggesting the involvement of TTF‐1 in cellular morphology and differentiation in SCLC cells. In addition, the molecular function terms included BH3/BH domain binding (Fig. S1C), indicating that TTF‐1 may associate with Bcl‐2 family genes, which regulate apoptosis. In contrast, TTF‐1‐bound genes in H441 cells were related to the MAP kinase pathway and cell‐to‐cell junction and no terms in the top 20 list were shared by the two cell lines (Fig. S1C).

### 3.3 TTF‐1 and ASCL1 bind to common genomic regions in SCLC cells

Gene expression profiling of SCLC cell lines revealed that the expression of *NKX2‐1* showed positive correlation with *ASCL1* but not with *NEUROD1*, whereas the expression of *MYC*, a target of NEUROD1, and *NKX2‐1* exhibited negative correlation (Fig. S2A). Likewise, *NKX2‐1* and *ASCL1* were coordinately expressed in SCLC tissue samples (http://www.ncbi.nlm.nih.gov/geo/query/acc.cgi?acc=GSE43346) (Fig. S2B). Investigations of the *ASCL1*, *NEUROD1*, and *MYC* expressions in several SCLC cell lines revealed a similar pattern (Fig. S2C). These findings suggest that expressions of TTF‐1 and ASCL1 are strongly related.

**Figure 3:**
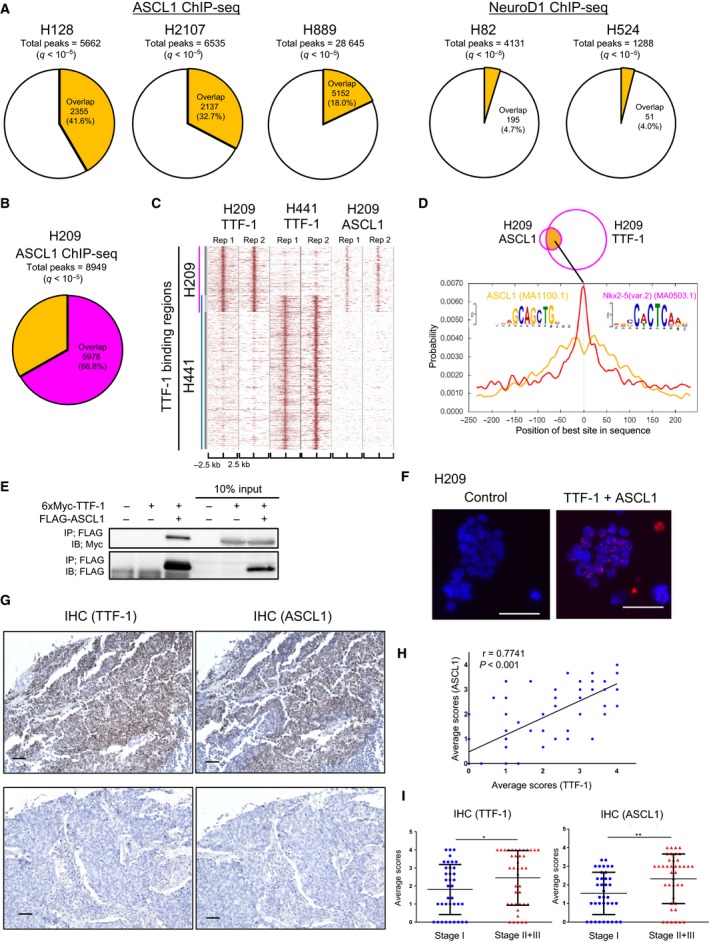
Interaction between TTF‐1 and ASCL1 proteins in SCLC. (A) Charts showing the overlaps with TTF‐1 binding regions in ASCL1 or NEUROD1 binding regions. The binding regions of ASCL1/NEUROD1 were identified by ChIP‐seq data of SCLC cell lines (http://www.ncbi.nlm.nih.gov/geo/query/acc.cgi?acc=GSE69398). (B) A chart showing the overlaps with TTF‐1 binding regions in ASCL1 binding regions in H209 cells. (C) A heat map representation of TTF‐1 and ASCL1 binding regions (two biological replicates, Rep 1 and Rep 2) in H441 and H209 cells. The vertical blue or magenta line indicates the TTF‐1 binding regions in H441 or H209 cells, respectively. (D) Motif centrality analysis using CentriMo in the overlapping regions between TTF‐1 and ASCL1 ChIP‐seq peaks. The 500‐bp sequences flanking the summit position of each TTF‐1 or ASCL1 binding region were used for the analysis. The known NKX‐homeodomain binding motif (Nkx2‐5, MA0503.1) and ASCL1 binding motif (ASCL1, MA1100.1) were used for calculation. The *x*‐axis indicates the relative position (bp) of the best site from the peak summit of each binding region. (E) Co‐IP assay of HEK293T cells transfected with expression plasmids for 6xMyc‐TTF‐1 and FLAG‐ASCL1. (F) *In situ* PLA using TTF‐1 and ASCL1 antibodies in H209 cells to show their proximity in the nucleus. H209 cells treated only with the TTF‐1 antibody were used as a control. Proximity between TTF‐1 and ASCL1 was detected as signals (red) in the nuclei (DAPI, blue). Scale bars, 50 µm. (G) Anti‐TTF‐1 and ASCL1 IHC on a tissue microarray of SCLC. The fraction of stained cancer cells was scored as shown in Fig. S4A. Representative images of TTF‐1‐ and ASCL1‐positive (upper panels, score 4) and negative (lower panels, score 0) tumors are shown. Scale bars, 50 µm. (H) Correlations of TTF‐1 and ASCL1 IHC staining scores in the SCLC tissue microarray. *r*, Spearman’s correlation coefficients. (I) Scatter plots of TTF‐1 (left) and ASCL1 (right) staining scores in a SCLC tissue microarray divided into two groups according to the SCLC stage. Data are represented as mean ± SE (*P < 0.05, **P < 0.01, Mann–Whitney *U*‐test).

Although the coexpression of TTF‐1 and ASCL1 and their binding motif‐based analyses have been reported in SCLC (Gazdar *et al.*, 2017; Park *et al.*, 2018), direct relationship between these TFs has not been fully investigated at a genome‐wide level. To this end, we obtained ASCL1 ChIP‐seq data from other SCLC cell lines (http://www.ncbi.nlm.nih.gov/geo/query/acc.cgi?acc=GSE69398). According to ASCL1 ChIP‐seq data in the ASCL1‐expressing cell lines (NCI‐H128, NCI‐H2107, and NCI‐H889) and NEUROD1 ChIP‐seq data in the NEUROD1‐expressing cell lines (NCI‐H82 and NCI‐H524), TTF‐1 binding regions in H209 cells had more overlap with the ASCL1 binding regions than with the NEUROD1 binding regions (Fig. 3A). We next carried out ASCL1 ChIP‐seq in H209 cells and identified 13 920 and 11 141 ASCL1 binding regions from each of the two biological replicates (*q* < 10^−5^). The 8949 common binding regions were then used in the following analyses. The known ASCL1 binding motif was significantly enriched and had a clear centrality (Fig. S3A), supporting the validity of the data. ASCL1 binding regions largely overlapped with the TTF‐1 binding regions in H209 cells but not with those in H441 cells (Fig. 3B,C). Moreover, motif centrality analysis in H209 cells showed unimodal distribution of the NKX‐homeodomain motif and bimodal distribution of the ASCL1 motif in the overlapping regions between TTF‐1 and ASCL1 ChIP‐seq peaks (Fig. 3D), suggesting that TTF‐1 and ASCL1 are closely located in the genomic DNA.

### 3.4 TTF‐1 physically and functionally interacts with ASCL1

We then found the physical interaction between TTF‐1 and ASCL1 using HEK293T cells ectopically expressing both TFs (Fig. 3E). Formation of the endogenous TTF‐1‐ASCL1 complex in H209 cells was also observed by *in situ* PLA to find their nuclear distribution (Fig. 3F). The predicted GO terms of the ASCL1‐bound genes were in part common to those of the TTF‐1‐bound genes, such as ‘BH domain binding’ and ‘aorta morphogenesis’ (Fig. S3B). Furthermore, *de novo* motif analysis in the overlapping regions between TTF‐1 and ASCL1 ChIP‐seq peaks identified both E‐box and NKX‐homeodomain motifs (Fig. S3C). These findings suggest that in SCLC cells, TTF‐1 interacts physically and functionally with ASCL1.

We also examined the relationship between TTF‐1 and ASCL1 expression in clinical SCLC tumors using a tissue array. IHC revealed that the expression of TTF‐1 and ASCL1 showed positive correlation in the nucleus of cancer cells (Fig. 3G,H and Fig. S4A). Additionally, both TTF‐1 and ASCL1 scores were significantly higher in advanced‐stage tumors than in earlier stage ones (Fig. 3I).

### 3.5 TTF‐1 and ASCL1 cooperatively regulate target gene expression

**Figure 4:**
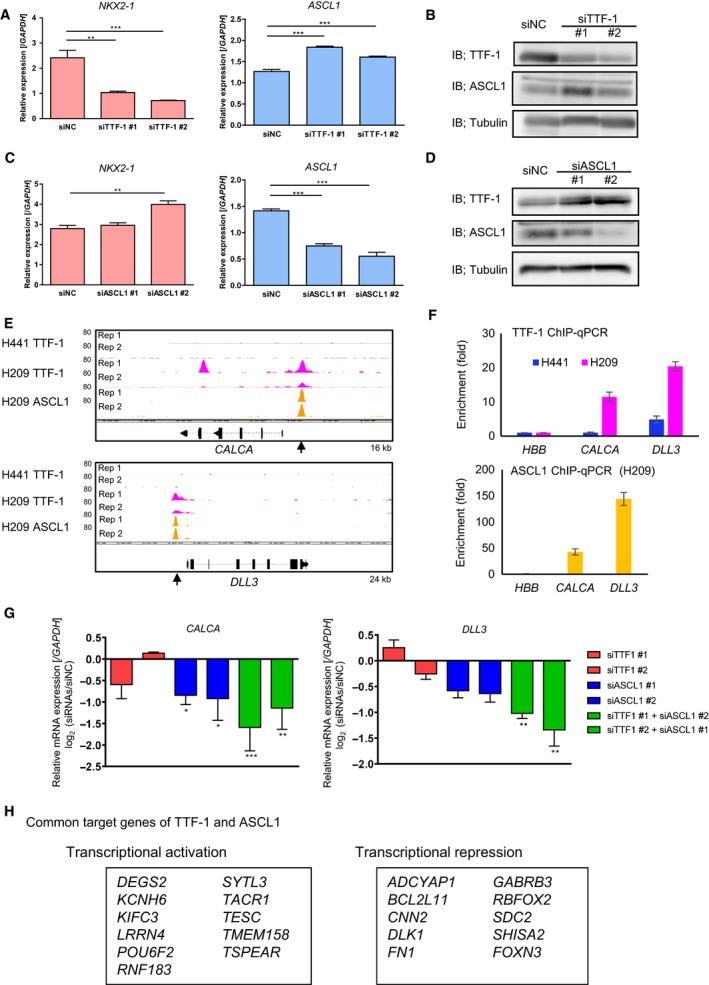
Cooperative regulation of the expression of target genes by TTF‐1 and ASCL1 in SCLC. (A) Expression of *NKX2‐1* (encoding TTF‐1; left) or *ASCL1* (right) mRNA in H209 cells treated with negative control (siNC) or TTF‐1 siRNAs (siTTF‐1) by qRT‐PCR. (B) IB for TTF‐1 and ASCL1 in H209 cells treated with siNC or siTTF‐1. (C) Expression of *NKX2‐1* and *ASCL1* mRNAs in H209 cells treated with siNC and ASCL1 siRNAs (siASCL1). (D) IB for TTF‐1 and ASCL1 in H209 cells treated with siNC or siASCL1. (E) Visualization of the TTF‐1 and ASCL1 ChIP‐seq data at the *CALCA* and *DLL3* gene loci. The upper two lanes show TTF‐1 binding signals in H441 cells. The middle or lower two lanes show TTF‐1 or ASCL1 binding signals in H209 cells, respectively. Rep 1 and Rep 2 indicate biological replicates 1 and 2. The kb sizes denote the ranges shown in the panels. Arrows show the position of ChIP‐qPCR analysis evaluated in (F). (F) ChIP‐qPCR analysis of TTF‐1 and ASCL1 at the genomic regions shown in (E). %input values at the target regions were normalized to those in *HBB* locus. (G) Fold change in the expression of *CALCA* and *DLL3* mRNA in H209 cells treated with siTTF‐1, siASCL1, or both, relative to those treated with siNC. Expression of mRNAs was quantified by qRT‐PCR. (H) Genes down‐ or upregulated by both TTF‐1 and ASCL1 in H209 cells. The genes with overlapping peaks between TTF‐1 and ASCL1 ChIP‐seq data were selected from the commonly regulated genes of TTF‐1 and ASCL1 determined by RNA‐seq of TTF‐1‐ or ASCL1‐depleted cells. These genes are listed in the boxes. qRT‐PCR data represented as mean ± SE of the three independent experiments. **P < 0.01, ***P < 0.001, one‐way ANOVA with Dunnett’s test.

Expression of TTF‐1 or ASCL1 was then silenced to evaluate their effects on target gene expression. We noticed that TTF‐1 knockdown led to ASCL1 upregulation (Fig. 4A,B), and ASCL1 knockdown led to upregulation of TTF‐1 protein in H209 cells (Fig. 4C,D). Although TTF‐1 and ASCL1 were coexpressed in SCLC (Fig. 3G,H), these findings suggested a tight regulation of the amounts of both TFs.

Significant TTF‐1 binding and ASCL1 binding were observed in the promoter region of *CALCA* (encoding CGRP) and *DLL3* genes (Fig. 4E,F), which are the genes characteristic of the neuroendocrine phenotype. We then conducted the knockdown experiments with siRNAs for TTF‐1 and ASCL1 using H209 cells (Fig. S5A,B) and assessed mRNA expression of these genes. The limited effect of each siRNA on the expression of *CALCA* and *DLL3* possibly reflected the mutual regulation between TTF‐1 and ASCL1, and enhanced inhibition of *CALCA* and *DLL3* expression was observed when both TTF‐1 and ASCL1 were depleted (Fig. 4G).

RNA‐seq was then performed following the knockdown of TTF‐1 or ASCL1 to find their targets in H209 cells (Fig. 4H). Some genes downregulated by both TTF1 and ASCL1 siRNAs were expressed in neuronal system, for example, *LRRN4*, *SYTL3*, *TACR1*, and *TESC*, suggesting that both TTF‐1 and ASCL1 were involved in the maintenance of neuroendocrine features in SCLC cells. One of the genes upregulated by both siRNAs was *BCL2L11* (encoding BIM) of the proapoptotic *BCL2* family gene. We confirmed that BIM protein was upregulated by TTF‐1 and/or ASCL1 knockdown in H209 cells (Fig. S6A,B).

### 3.6 TTF‐1 positively regulates the expression of Bcl‐2

**Figure 5:**
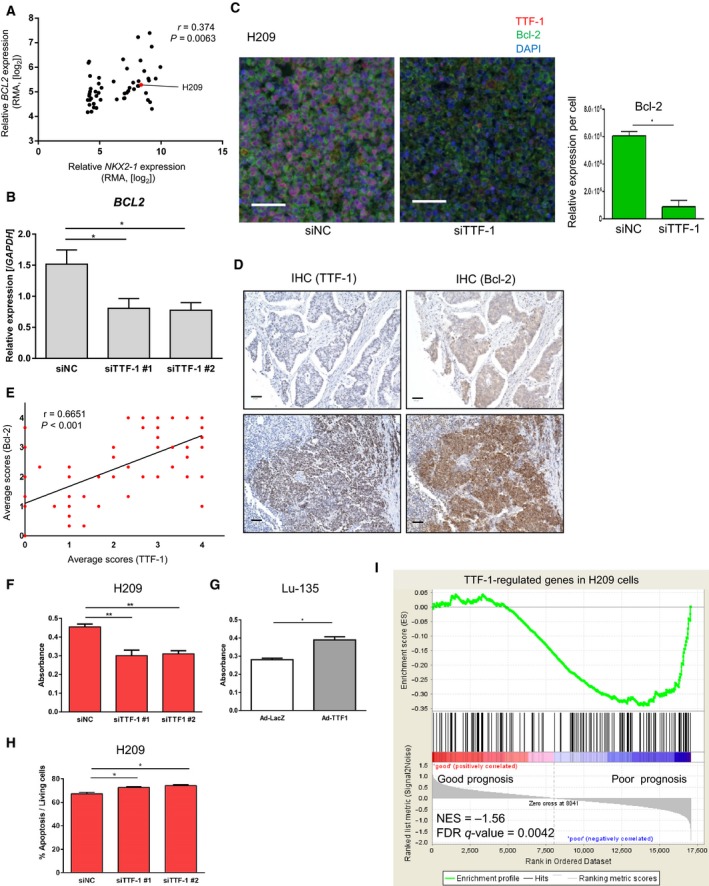
Regulation of cell growth and *BCL2* by TTF‐1 in SCLC. (A) Correlations of *NKX2‐1* and *BCL2* gene expressions in SCLC cells of the CCLE database. *r*, Spearman’s correlation coefficients. (B) Relative expression of *BCL2* mRNA in H209 cells treated with siTTF‐1 relative to those treated with siNC. (C) Immunofluorescence staining for TTF‐1 (red) and Bcl‐2 (green) in H209 cells treated with siNC (left) or siTTF‐1 #2 (right). Right panel shows the result of quantification of the Bcl‐2 expression. Data represented as mean ± S.E of randomly selected two microscopic fields. *P < 0.05, unpaired *t*‐test. Scale bars, 50 µm. (D) Anti‐TTF‐1 (left) and Bcl‐2 (right) IHC on a tissue microarray of SCLC. The intensity of staining was scored as shown in Fig. S4B. Representative images are shown. Scale bars, 50 µm. (E) Correlations of TTF‐1 and Bcl‐2 staining scores in the SCLC tissue microarray. *r*, Spearman’s correlation coefficients. (F) WST‐8 cell proliferation assay in H209 cells treated with siNC or siTTF‐1. (G) WST‐8 cell proliferation assay in Lu‐135 cells infected with adenovirus for LacZ (Ad‐LacZ) and TTF‐1 (Ad‐TTF1) expression. *P < 0.05, unpaired *t*‐test. (H) Induction of apoptosis by knockdown of TTF‐1 in H209 cells. Annexin V‐positive cells were evaluated by flow cytometry. *P < 0.05, one‐way ANOVA with Dunnett’s test. (I) Enrichment plot of the TTF‐1‐regulated gene set (fold‐changes ≤ 0.66) in H209 cells. GSEA was performed using the gene expression data of the clinical SCLC samples (http://www.ncbi.nlm.nih.gov/geo/query/acc.cgi?acc=GSE43346). Patients’ samples were divided into good (*n* = 12) and poor (*n* = 11) prognosis groups as in Fig. 1D. NES, normalized enrichment score. Data represented as mean ± S.E of the three (B, F, H) or two (G) independent experiments. *P < 0.05, **P < 0.01, one‐way ANOVA with Dunnett’s test.

We further focused on *BCL2* expression as a TTF‐1‐bound target gene. Gene expression profiling of SCLC cell lines in CCLE dataset revealed that *BCL2* exhibited positive correlation with *NKX2‐1* (Fig. 5A). Moreover, TTF‐1 knockdown downregulated the mRNA expression of *BCL2* in H209 cells (Fig. 5B). We also confirmed that knockdown of TTF‐1 decreased the Bcl‐2 expression by the immunofluorescent assay in H209 cells (Fig. 5C). IHC of a SCLC tissue array showed the expression of TTF‐1 positively correlated with Bcl‐2 (Fig. 5D,E and Fig. S4B). Although ASCL1 also reportedly upregulates *BCL2* in some of the SCLC, the effect of ASCL1 siRNA on *BCL2* was not remarkable in H209 cells (data not shown). These results suggest a complementary role of TTF‐1 in the regulation of *BCL2*.

### 3.7 TTF‐1 promotes survival of SCLC cells and regulates the genes associated with poor prognosis

We postulated that TTF‐1 promotes survival of SCLC cells through inhibition of apoptosis, similar to ASCL1 (Murai *et al.*, 2015). Using WST‐8 assay, we revealed that TTF‐1 silencing in H209 cells resulted in decreased cell viability (Fig. 5F). On the contrary, ectopic TTF‐1 by adenovirus vector increased cell viability in TTF‐1‐low Lu‐135 cells (Fig. 5G and Fig. S7). We further investigated whether TTF‐1 knockdown could enhance apoptosis of H209 cells. As a nature of SCLC cell lines, baseline population of apoptotic cells in the culture was high (Horie *et al.*, 2018). However, the fractions of Annexin‐positive apoptotic cells marginally but significantly increased after TTF‐1 knockdown (Fig. 5H).

Finally, we examined enrichment of 166 genes, which were downregulated by TTF‐1 knockdown in H209 cells, using expression arrays of clinical SCLC samples (http://www.ncbi.nlm.nih.gov/geo/query/acc.cgi?acc=GSE43346) to explore its relationship to patient prognosis. When this SCLC cohort was divided into good and poor prognosis groups, TTF‐1‐regulated genes were significantly enriched in the poor prognosis group (Fig. 5I), which also suggested the tumor‐promoting role of TTF‐1 in clinical SCLC tumors.

## 4 Discussion

In this study, we clarified the distinct properties of TTF‐1 binding regions between SCLC and LADC. Our findings suggested that TTF‐1 promotes SCLC growth and contributes to neural differentiation by partly coordinating with ASCL1. One of the representative motifs commonly enriched in the TTF‐1 binding regions in both H441 and H209 cells was that of the Forkhead family genes. Forkhead family TFs are pioneer factors that target enhancers for tissue‐specific gene activation during development and cellular reprogramming (Iwafuchi‐Doi *et al.*, 2016). In a murine LADC model, TTF‐1 physically binds and interacts with FOXA, providing a direct connection between transcriptional lung differentiation programs and tumor initiation (Snyder *et al.*, 2013). Therefore, the differential TTF‐1 binding regions between SCLC and LADC appear to be determined epigenetically by Forkhead TFs.

Previous comprehensive genomic studies indicate that SCLC harbors several somatic mutations, such as deletion of the *TP53* and *RB1* genes (Peifer *et al.*, 2012). Moreover, several TF genes are amplified, including *MYC*, *NFIB*, and *SOX2* (George *et al.*, 2015; Kim *et al.*, 2006; Rudin *et al.*, 2012). Among them, ASCL1 is believed to be a key regulator of neuroendocrine differentiation (Borges *et al.*, 1997; Osada *et al.*, 2008) and a lineage‐survival oncogene of SCLC (Borromeo *et al.*, 2016). ASCL1 is also known as a repressive target of Smad TFs downstream of transforming growth factor‐β (TGF‐β) and is required for tumor formation by suppressing apoptosis in SCLC cells (Murai *et al.*, 2015). ASCL1, insulinoma‐associated 1 (INSM1) zinc finger transcription factor, and BRN2 collaborate to form regulatory circuitry involved in neuroendocrine differentiation of SCLC (Borromeo *et al.*, 2016; Fujino *et al.*, 2015; Sakaeda *et al.*, 2013). Furthermore, concomitant enrichment of proneural TFs (including ASCL1) and NKX‐homeodomain TFs (including TTF‐1) is critical for transformation to small‐cell neuroendocrine carcinoma (Park *et al.*, 2018). Consistently, our genome‐wide analysis of SCLC cells revealed that TTF‐1 binding regions and TTF‐1‐regulated genes are associated with cellular differentiation and neural development. Of note, LADC that expresses ASCL1 shows neuroendocrine phenotype (Miyashita *et al.*, 2018). Other reports indicate that LADC transforms into SCLC during the course of clinical treatments (Ferrer *et al.*, 2019; Marcoux *et al.*, 2019). In LADC cells, TTF‐1 interacts with the Smad family TFs downstream of TGF‐β signaling to inhibit EMT, providing a mechanism of tumor suppressor function of TTF‐1 (Isogaya *et al.*, 2014). TGF‐β signaling is frequently silenced in SCLC (Murai *et al.*, 2015). The study of dynamics of changes in genomic distribution of TTF‐1 and its co‐TFs, during the transformation of LADC into SCLC, could help clarify its complex roles during tumor progression and differentiation.

Bcl‐2, one of the transcriptional targets of ASCL1, is known as an antiapoptotic regulator and acts as an oncogene in neuroendocrine lung cancers (Augustyn *et al.*, 2014). Our results revealed that TTF‐1 positively regulated the expression of Bcl‐2 in SCLC cells and was coexpressed in clinical tissues. Bcl‐2 expression can be presumably enhanced by TTF‐1 in clinical tumors, which in turn may participate in SCLC progression. Consistently, Cardnell and colleagues have reported the association between TTF‐1 expression and sensitivity to a Bcl‐2 inhibitor (Cardnell *et al.*, 2017). Considering the cooperative regulation of proapoptotic BIM expression, relationship between TTF‐1 and ASCL1 might be of special clinical significance as a predictive marker of SCLC treatment.

## 5 Conclusions

Our results revealed distinct properties of TTF‐1 distribution on the genome in SCLC. Our genome‐wide analysis unraveled different roles of TTF‐1 between LADC and SCLC and revealed its transcriptional regulatory programs related to antiapoptotic and neuroendocrine gene expression in SCLC.

## Conflict of interest

KM and SE were partly supported by Eisai, Co., Ltd. The remaining authors declare no conflict of interest.

## Author contributions

SH performed most of the in vitro experiments together with YT and A Katsura. SH performed the immunohistochemistry. FM and SE determined the experimental conditions of the SCLC cell lines. SH, YT, A Kaneda, MM, ST, HA, and DK acquired and analyzed the high‐throughput sequencing data. SH, DK, and KM conceived and designed the project. A Kaneda, YI, and HA obtained and analyzed the patient samples. SH, DK, and KM wrote the manuscript.

## Supporting information


**Fig. S1.** Comparison of TTF‐1 ChIP‐seq data in H441 and H209 cells.
**Fig. S2.** Positive correlation between *NKX2‐1* and *ASCL1* expression in small cell lung cancer cell lines and tissue samples.
**Fig. S3.** Characteristics of ASCL1 ChIP‐seq data in H209 cells.
**Fig. S4.** Immunohistochemistry (IHC) scores of TTF‐1 and Bcl‐2.
**Fig. S5.** Validation of ASCL1 siRNA and double knockdown with TTF‐1 and ASCL1 siRNAs.
**Fig. S6.** Regulation of BIM expression by TTF‐1 and ASCL1 in H209 cells.
**Fig. S7.** Validation of TTF‐1 adenoviral expression vector.

**Table S1.** Primer sequences for qRT‐PCR analyses of human mRNA.
**Table S2.** Primer sequences for ChIP‐qPCR analyses.

**Data S1.** Output data of the motif analysis of TTF‐1 binding regions in H209 cells using DREME, supporting data for Figure 2F.

**Data S2.** Output data of the motif analysis of TTF‐1 binding regions in H441 cells using DREME, supporting data for Figure 2F.

**Data S3.** Output data of the motif analysis of TTF‐1‐ASCL1 co‐binding regions in H209 cells using DREME, supporting data for Figure S3C.

## Data Availability

The raw ChIP‐seq and RNA‐seq data have been deposited to GEO (http://www.ncbi.nlm.nih.gov/geo/query/acc.cgi?acc=GSE129341).
