# Supplementary material for: Comparative analysis of TTF‐1 binding DNA regions in small‐cell lung cancer and non‐small‐cell lung cancer
Source: Mol Oncol. 2019 Dec 15;14(2):277–93. doi: 10.1002/1878-0261.12608 (PMC6998394; doi:10.1002/1878-0261.12608)

Figure S1

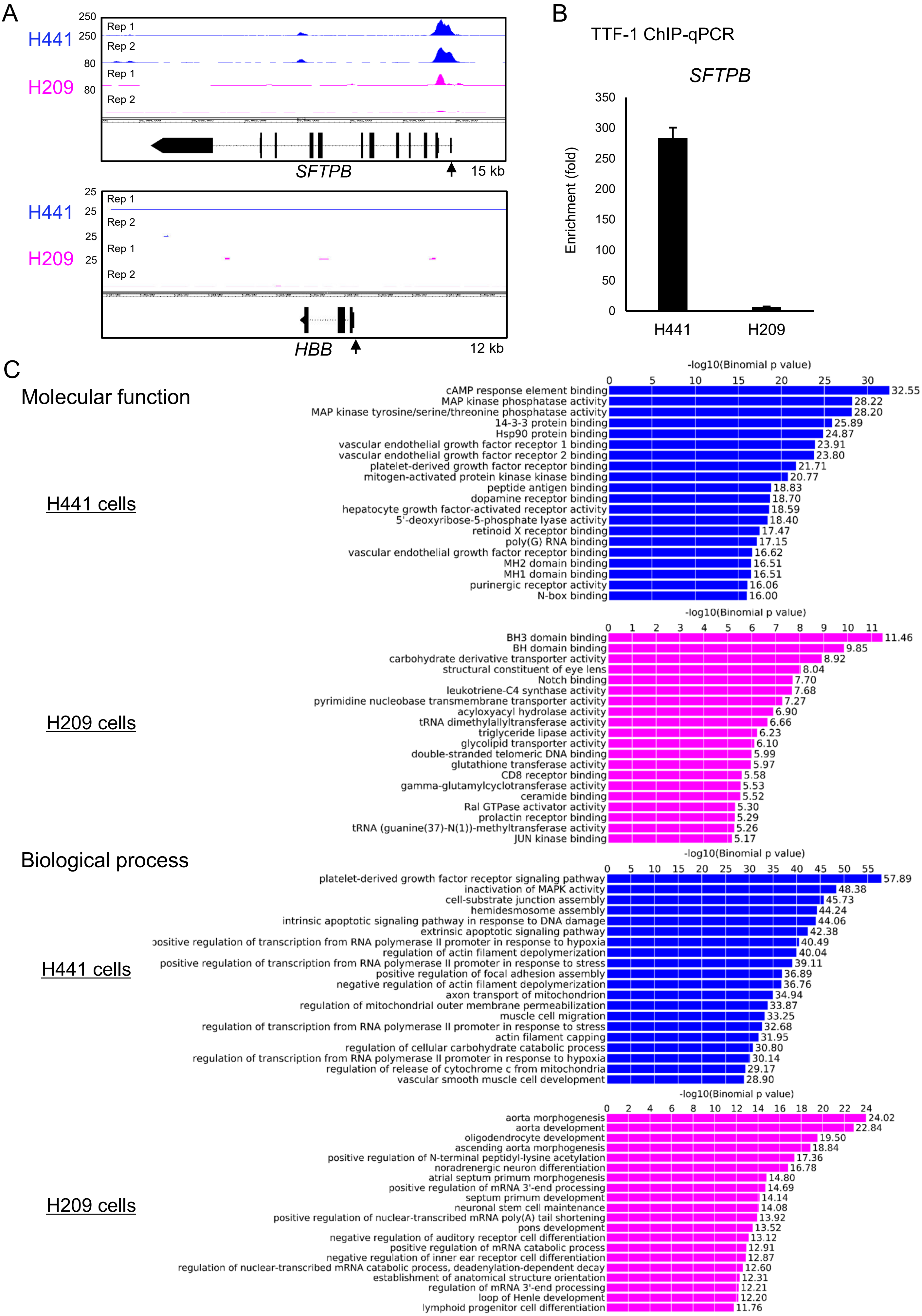
A
H441
H209
Rep 1
Rep 2
Rep 1
Rep 2
250
250
80
80
SFTPB
15 kb
25
25
25
25
HBB
12 kb
B
TTF-1 ChIP-qPCR
SFTPB
Enrichment (fold)
0
50
100
150
200
250
300
350
H441
H209
C
Molecular function
H441 cells
-log10(Binomial p value)
cAMP response element binding 32.55
MAP kinase phosphatase activity 28.22
MAP kinase tyrosine/serine/threonine phosphatase activity 28.20
14-3-3 protein binding 25.89
Hsp90 protein binding 24.87
vascular endothelial growth factor receptor 1 binding 23.91
vascular endothelial growth factor receptor 2 binding 23.80
platelet-derived growth factor receptor binding 21.71
mitogen-activated protein kinase kinase binding 20.77
peptide antigen binding 18.83
dopamine receptor binding 18.70
hepatocyte growth factor-activated receptor activity 18.59
5'-deoxyribose-5-phosphate lyase activity 18.40
retinoid X receptor binding 17.47
poly(G) RNA binding 17.15
vascular endothelial growth factor receptor binding 16.62
MH2 domain binding 16.51
MH1 domain binding 16.51
purinergic receptor activity 16.06
N-box binding 16.00
H209 cells
-log10(Binomial p value)
BH3 domain binding 11.46
BH domain binding 9.85
carbohydrate derivative transporter activity 8.92
structural constituent of eye lens 8.04
Notch binding 7.70
leukotriene-C4 synthase activity 7.68
pyrimidine nucleobase transmembrane transporter activity 7.27
acyloxyacyl hydrolase activity 6.90
tRNA dimethylallyltransferase activity 6.66
triglyceride lipase activity 6.23
glycolipid transporter activity 6.10
double-stranded telomeric DNA binding 5.99
glutathione transferase activity 5.97
CD8 receptor binding 5.58
gamma-glutamylcyclotransferase activity 5.53
ceramide binding 5.52
Ral GTPase activator activity 5.30
prolactin receptor binding 5.29
tRNA (guanine(37)-N(1))-methyltransferase activity 5.26
JUN kinase binding 5.17
Biological process
H441 cells
-log10(Binomial p value)
platelet-derived growth factor receptor signaling pathway 57.89
inactivation of MAPK activity 48.38
cell-substrate junction assembly 45.73
hemidesmosome assembly 44.24
intrinsic apoptotic signaling pathway in response to DNA damage 44.06
extrinsic apoptotic signaling pathway 42.38
positive regulation of transcription from RNA polymerase II promoter in response to hypoxia 40.49
regulation of actin filament depolymerization 40.04
positive regulation of transcription from RNA polymerase II promoter in response to stress 39.11
positive regulation of focal adhesion assembly 36.89
negative regulation of actin filament depolymerization 36.76
axon transport of mitochondrion 34.94
regulation of mitochondrial outer membrane permeabilization 33.87
muscle cell migration 33.25
regulation of transcription from RNA polymerase II promoter in response to stress 32.68
actin filament capping 31.95
regulation of cellular carbohydrate catabolic process 30.80
regulation of transcription from RNA polymerase II promoter in response to hypoxia 30.14
regulation of release of cytochrome c from mitochondria 29.17
vascular smooth muscle cell development 28.90
H209 cells
-log10(Binomial p value)
aorta morphogenesis 24.02
aorta development 22.84
oligodendrocyte development 19.50
ascending aorta morphogenesis 18.84
positive regulation of N-terminal peptidyl-lysine acetylation 17.36
noradrenergic neuron differentiation 16.78
atrial septum primum morphogenesis 14.80
positive regulation of mRNA 3'-end processing 14.69
septum primum development 14.14
neuronal stem cell maintenance 14.08
positive regulation of nuclear-transcribed mRNA poly(A) tail shortening 13.92
pons development 13.52
negative regulation of auditory receptor cell differentiation 13.12
positive regulation of mRNA catabolic process 12.91
negative regulation of inner ear receptor cell differentiation 12.87
regulation of nuclear-transcribed mRNA catabolic process, deadenylation-dependent decay 12.60
establishment of anatomical structure orientation 12.31
regulation of mRNA 3'-end processing 12.21
loop of Henle development 12.20
lymphoid progenitor cell differentiation 11.76

Figure S2

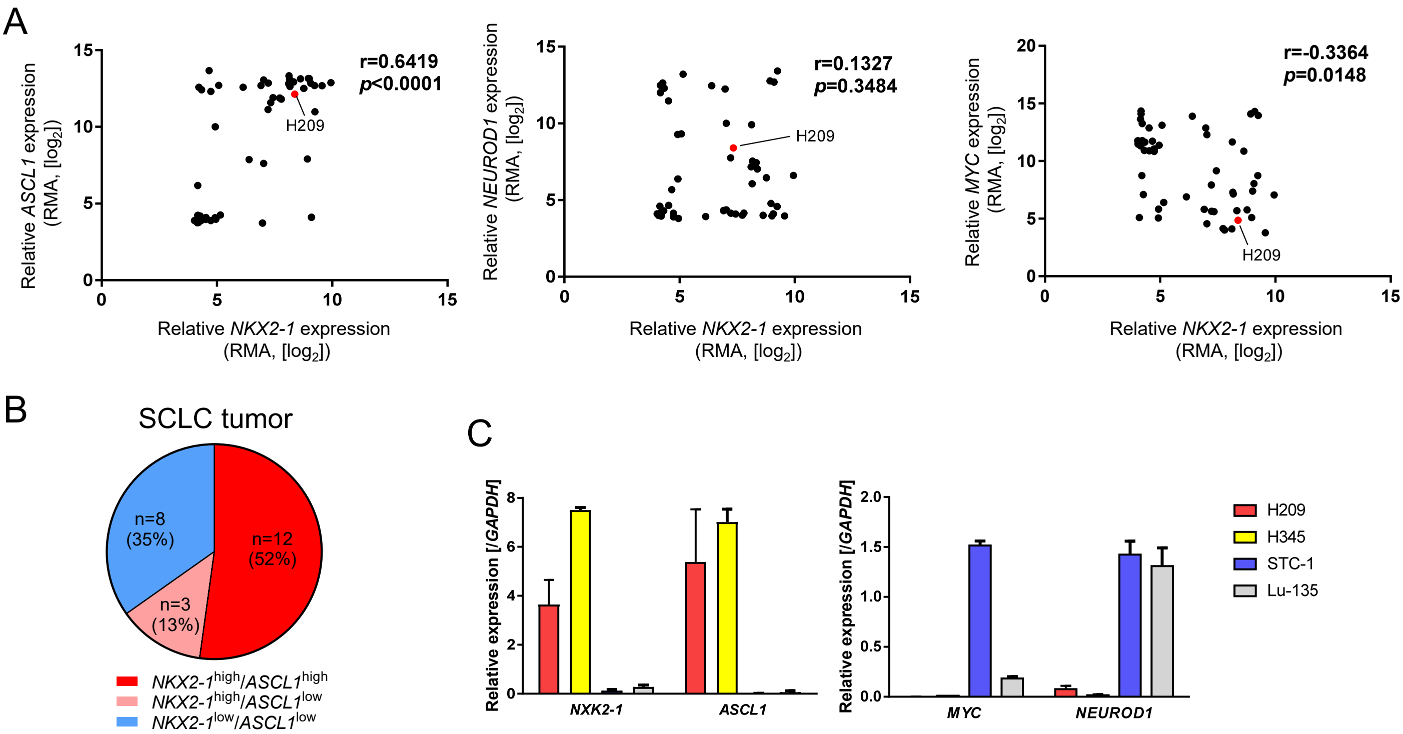
A
r=0.6419
p<0.0001
H209
Relative ASCL1 expression (RMA, [log2])
Relative NKX2-1 expression (RMA, [log2])
r=0.1327
p=0.3484
Relative NEUROD1 expression (RMA, [log2])
r=-0.3364
p=0.0148
Relative MYC expression (RMA, [log2])
B
SCLC tumor
n=8 (35%)
n=12 (52%)
n=3 (13%)
NKX2-1high/ASCL1high
NKX2-1high/ASCL1low
NKX2-1low/ASCL1low
C
Relative expression [/GAPDH]
NXK2-1
ASCL1
MYC
NEUROD1
H209
H345
STC-1
Lu-135

Figure S3

A

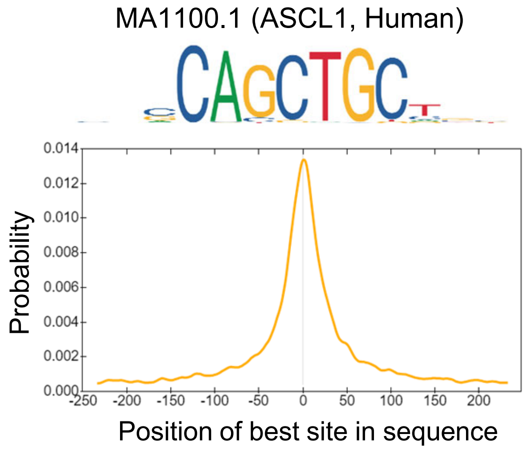

B

Molecular function

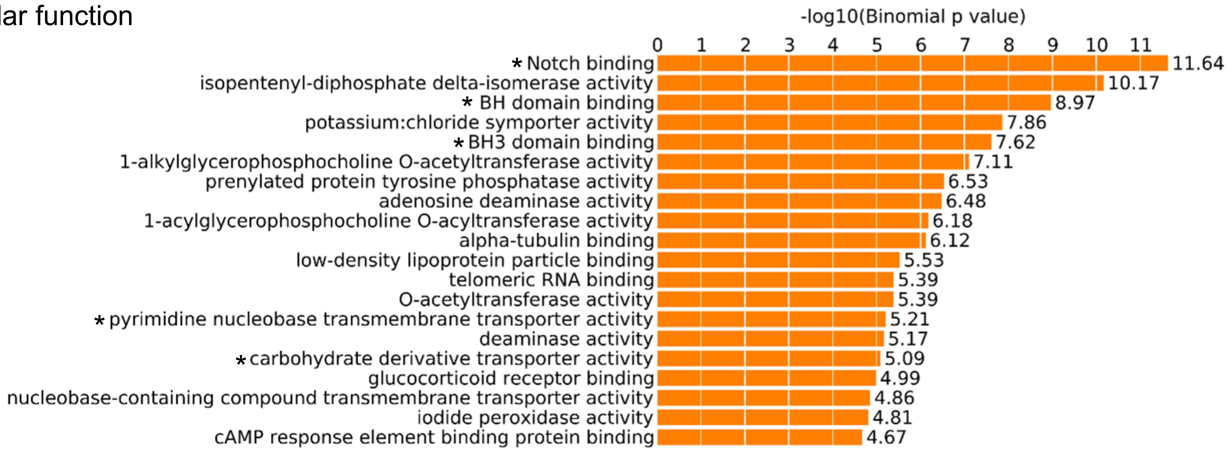

Biological process

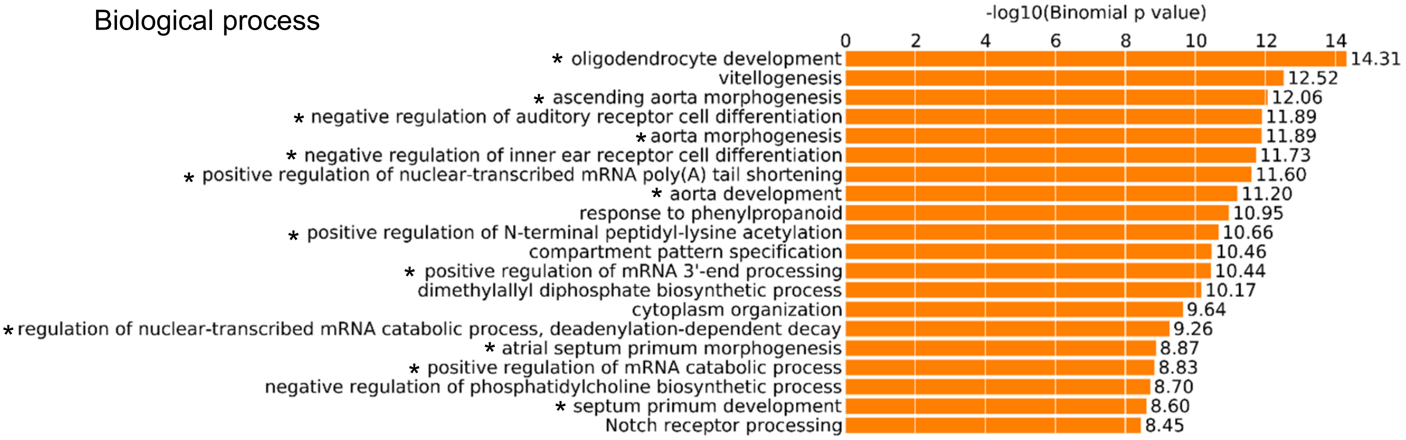

C

Cluster	Top motifs (E-value)	Similar motif	Distribution	Matched sequence (%) (CentriMo Group)
1	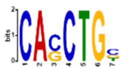 (6.2e-433)	E-box	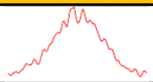	86%
2	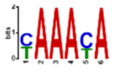 (7.1e-119)	Forkhead	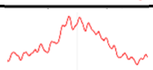	79%
3	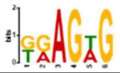 (8.2e-53)	NKX-homeodomain	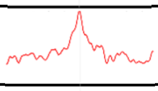	30%

Figure S4

A

IHC（TTF-1)

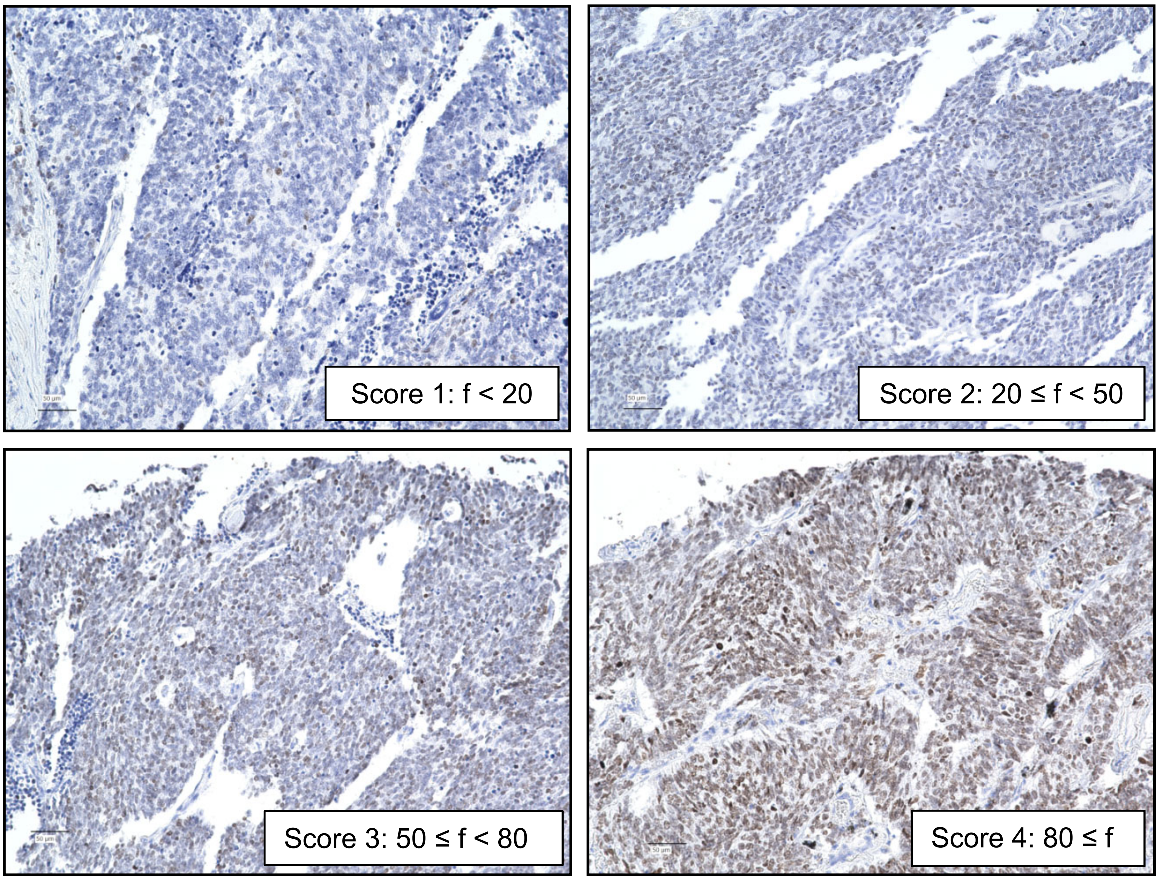

B

IHC (Bcl-2)

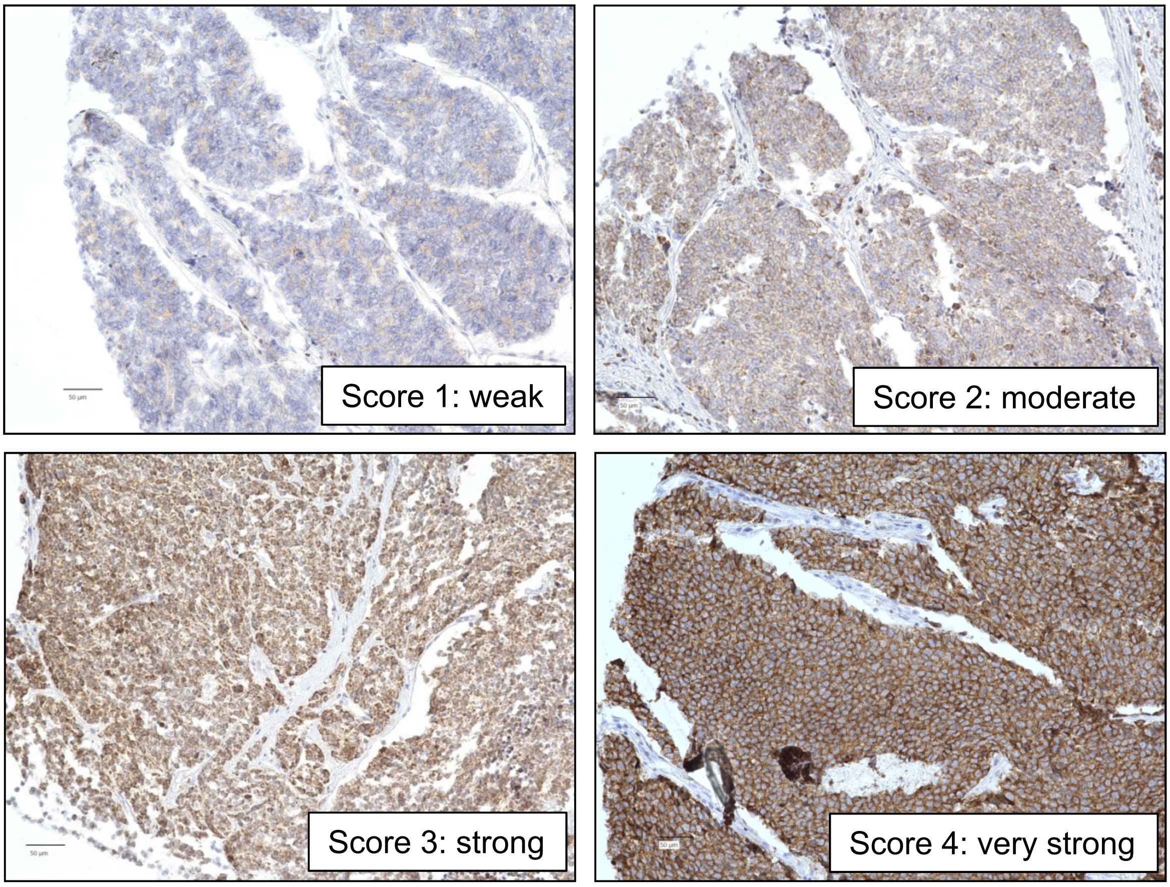

Figure S5

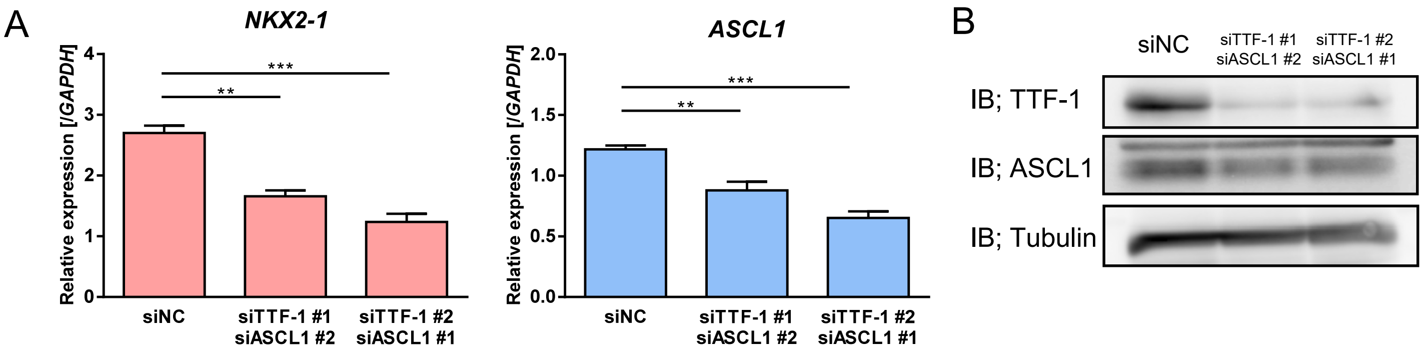
A
NKX2-1
Relative expression [/GAPDH]
0
1
2
3
4
**
***
siNC
siTTF-1 #1
siASCL1 #2
siTTF-1 #2
siASCL1 #1
ASCL1
Relative expression [/GAPDH]
0.0
0.5
1.0
1.5
2.0
**
***
siNC
siTTF-1 #1
siASCL1 #2
siTTF-1 #2
siASCL1 #1
B
siNC
siTTF-1 #1
siASCL1 #2
siTTF-1 #2
siASCL1 #1
IB; TTF-1
IB; ASCL1
IB; Tubulin

Figure S6

A

B

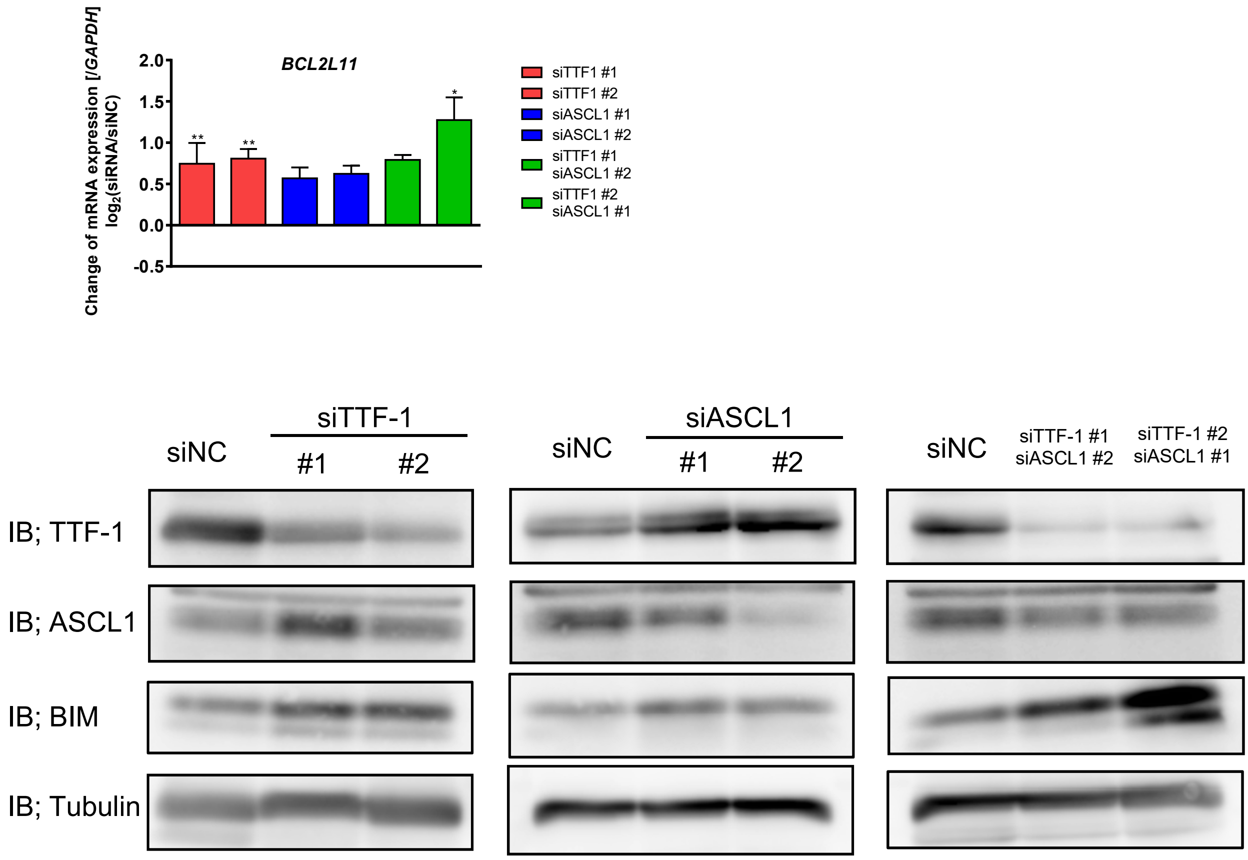
BCL2L11
Change of mRNA expression [/GAPDH]
log2(siRNA/siNC)
2.0
1.5
1.0
0.5
0.0
-0.5
**
**
*
siTTF1 #1
siTTF1 #2
siASCL1 #1
siASCL1 #2
siTTF1 #1
siASCL1 #2
siTTF1 #2
siASCL1 #1
siNC
siTTF-1
#1
#2
siNC
siASCL1
#1
#2
siNC
siTTF-1 #1
siASCL1 #2
siTTF-1 #2
siASCL1 #1
IB; TTF-1
IB; ASCL1
IB; BIM
IB; Tubulin

Figure S7

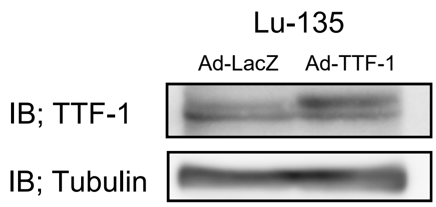

Supplement: Supplementary file 1 — Fig. S1. Comparison of TTF‐1 ChIP‐seq data in H441 and H209 cells. Fig. S2. Positive correlation between NKX2‐1 and ASCL1 expression in small cell lung cancer cell lines and tissue samples. Fig. S3. Characteristics of ASCL1 ChIP‐seq data in H209 cells. Fig. S4. Immunohistochemistry (IHC) scores of TTF‐1 and Bcl‐2. Fig. S5. Validation of ASCL1 siRNA and double knockdown with TTF‐1 and ASCL1 siRNAs. Fig. S6. Regulation of BIM expression by TTF‐1 and ASCL1 in H209 cells. Fig. S7. Validation of TTF‐1 adenoviral expression vector. [file MOL2-14-277-s001.pdf]
