# Supplementary material for: Comparative analysis of TTF‐1 binding DNA regions in small‐cell lung cancer and non‐small‐cell lung cancer
Source: Mol Oncol. 2019 Dec 15;14(2):277–93. doi: 10.1002/1878-0261.12608 (PMC6998394; doi:10.1002/1878-0261.12608)
Supplement: Supplementary file 2 — Table S1. Primer sequences for qRT‐PCR analyses of human mRNA. Table S2. Primer sequences for ChIP‐qPCR analyses. [file MOL2-14-277-s002.docx]

**Supporting Information**

**Table S1. Primer sequences for qRT-PCR analyses of human mRNA.**

| **Gene** | **Orientation** | **Sequence (5' to 3')** |
| --- | --- | --- |
| *GAPDH* | Forward | GAAGGTGAAGGTCGGAGTC |
|  | Reverse | GAAGATGGTGATGGGATTTC |
| *NKX2-1* | Forward | CAGCAAAGAGGACTCGCTTGT |
|  | Reverse | GGCACTTCCTTTGTGTCAGTGA |
| *ASCL1* | Forward | TCTTACGACCCGCTCAGCCCC |
|  | Reverse | AGGTTGTGCGATCACCCTGCTT |
| *CALCA* | Forward | TCTAAGCGGTGCGGTAATCTG |
|  | Reverse | CAGTTTGGGGGAACGTGTGA |
| *DLL3* | Forward | CAACTGTGAGAAGAGGGTGGA |
|  | Reverse | CCAGGTCCAGGCAGAGTC |
| *BCL2* | Forward | ATGTGTGTGGAGAGCGTCAA |
|  | Reverse | GGGCCGTACAGTTCCACAAA |
| *MYC* | Forward | CCACACATCAGCACAACTACGC |
|  | Reverse | CGGTTGTTGCTGATCTGTCTCA |
| *NEUROD1* | Forward | TCTTCCACGTTAAGCCTCCG |
|  | Reverse | CCATCAAAGGAAGGGCTGGT |
| *BCL2L11* | Forward | ATCTCAGTGCAATGGCTTCC |
|  | Reverse | CAACTCTTGGGCGATCCATA |

**Table S2. Primer sequences for ChIP-qPCR analyses.**

| **Gene** | **Orientation** | **Sequence (5' to 3')** |
| --- | --- | --- |
| *HBB* | Forward | AGATGGCTCTGCCCTGACTT |
|  | Reverse | AAGAGCCAAGGACAGGTACG |
| *SFTPB* | Forward | AGCGACCTCAGTGTTTGTCT |
|  | Reverse | GCTGGGAAAAGGTGGGATCA |
| *KRT4* | Forward | TCAACTTGGCCAACAGCTACT |
|  | Reverse | GAGGTGTTTTGTCTTCCCCCA |
| *SDPR* | Forward | ATTGACACGTGCCCAGAAGT |
|  | Reverse | AGGTGTGCCACTTCACTGAG |
| *NFIB* | Forward | ACAGGCACACTGGGTCATTT |
|  | Reverse | GCACAGGTGCAGCATGTAGT |
| *NCAM1* | Forward | TTTCCCCTGAGACAGTGTGC |
|  | Reverse | AAACCTCTCCTGCAGCCCTA |
| *CALCA* | Forward | CTACCCTGCCATCCATCACC |
|  | Reverse | ACATCTTGGCAAACAGCAGC |
| *DLL3* | Forward | ACACCTTCCGGAGGCCAAAC |
|  | Reverse | GGGGCTCCCAGCTGTATGTAA |

**Supplementary Figure Legends**

**Figure S1.** **Comparison of TTF-1 ChIP-seq data in H441 and H209 cells**

**(A)** TTF-1 binding signals at the *SFTPB* and *HBB* gene loci. The upper or lower two lanes show TTF-1 binding signals from NCI-H441 (H441) (blue) or NCI-H209 (H209) (magenta) cells, respectively. The y-axis scales are not equal as they were adjusted to show the binding signals clearly for each lane. The kb sizes denote the ranges shown in the panels. The arrows show the genomic positions evaluated by ChIP-qPCR in (B). **(B)** ChIP-qPCR analysis of TTF-1 binding at the *SFTPB* locus. Enrichment was calculated by normalizing %input values at *SFTPB* locus to those at *HBB* locus and were represented as mean ± S.E of the biological replicates (n=2). **(C)** Gene ontology terms of molecular function or biological process determined by the TTF-1-bound genes, which were annotated by GREAT ver 3.0.0 using the genomic regions of TTF-1 ChIP-seq peaks. The terms were sorted by *P*-values. Values indicate -log_10_ (*P*-value).

**Figure S2.** **Positive correlation between *NKX2-1* and *ASCL1* expression in small cell lung cancer cell lines and tissue samples**

**(A)** Correlations of *NKX2-1* gene expression with *ASCL1* (left), *NEUROD1* (middle), and *MYC* (right) expression in SCLC cell lines. The gene expression data were obtained from CCLE database. Spearman correlation coefficients (r) and *P*-values are indicated. **(B)** A chart showing the proportion of three SCLC tumor groups classified by the expression level of *NKX2-1* and *ASCL1* gene expression. The expression data were obtained by microarray from 23 clinical SCLC tumor samples (GSE43346). High expression was defined as Genechip Score > 100. **(C)** Expression of *NKX2-1*, *ASCL1*, *MYC*, and *NEUROD1* mRNAs in SCLC cell lines quantified by qRT-PCR analysis. Data represented as mean± S.E (n=3).

**Figure S3.** **Characteristics of ASCL1 ChIP-seq data in H209 cells**

**(A)** Motif centrality analysis using CentriMo version 5.0.5, performed from the 500 bp sequences flanking the summit position of each ASCL1 binding region. The known ASCL1 motif model from JASPAR (ASCL1, MA1100.1) was used for calculation. The x-axis indicates the relative position (bp) of the best site from the peak summit of each binding region. **(B)** Gene ontology terms of biological process or molecular function determined from the ASCL1 binding genes, which were annotated by GREAT ver 3.0.0 using the genomic regions of ASCL1 ChIP-seq peaks in H209 cells. The terms were sorted by *P*-values. Values indicate -log_10_ (*P*-value). The terms with asterisks indicate overlap with those determined by TTF-1 binding genes (Fig. S1C). **(C)** Motif enrichment and centrality analysis using DREME version 5.0.5 and CentriMo version 5.0.5, respectively. The sequences used for the calculation are the overlap regions between TTF-1 and ASCL1 binding genomic regions in H209 cells. The top three *de novo* motifs with lowest E-values are shown.

**Figure S4.** **Immunohistochemistry (IHC) scores of TTF-1 and Bcl-2**

**(A)** Images representing TTF-1 IHC scores in a tissue microarray of SCLC. The fraction of stained tumor cells was scored as follows. 0: 0%, 1: 1%-20%, 2: 21%-50%, 3: 51%-80%, 4: > 81%. **(B)** Images representing Bcl-2 IHC scores in a tissue microarray of SCLC. The intensity of staining was scored as follows. 0: negative, 1: weak, 2: moderate, 3: strong, 4: very strong. Scale bars, 50 μm.

**Figure S5.** **Validation of ASCL1 siRNA and double knockdown with TTF-1 and ASCL1 siRNAs**

**(A)** Expression of *NKX2-1* and *ASCL1* mRNAs in H209 cells treated with siNC or both (TTF-1 siRNA (siTTF-1) and siASCL1) quantified by qRT-PCR analysis. **(B)** Immunoblotting (IB) for TTF-1 and ASCL1 in H209 cells treated with siNC or both (siTTF-1 and siASCL1). Data represented as mean± S.E of the three independent experiments; ** *P* < 0.01, *** *P* < 0.001; one-way ANOVA with Dunnett’s test.

**Figure S6. Regulation of BIM expression by TTF-1 and ASCL1 in H209 cells**

**(A)** Fold change of *BCL2L11* (encoding BIM) mRNA expression in NCI-H209 (H209) cells treated with siRNA for TTF-1 (siTTF-1), for ASCL1 (siASCL1), or both (siTTF-1 and siASCL1) relative to those treated with negative control siRNA (siNC). Values of mRNA were quantified by qRT-PCR analysis. Data represent mean number of the biological triplicates. Error bars indicate the S.E. * *P* < 0.05, ** *P* < 0.01; one-way ANOVA with Dunnett’s test. NC, negative control. **(B)** Immunoblotting for TTF-1, ASCL1, and BIM in H209 cells treated with siNC, siTTF-1, or siASCL1. Note that the same samples were used in Fig. 4B, D, and Fig. S5B, and the same anti-TTF1, anti-ASCL1, and anti-Tubulin blots were shown as references.

**Figure S7. Validation of TTF-1 adenoviral expression vector**

Immunoblotting (IB) for TTF-1 in Lu-135 cells infected with adenovirus for LacZ (Ad-LacZ) and TTF-1 (Ad-TTF1).

**Supporting Data S1. Output data of the motif analysis of TTF-1 binding regions in H209 cells using DREME, supporting data for Figure 2F**

500 bp sequences franking the peak summit position of TTF-1 binding regions in H209 cells were used for motif analysis by DREME.

**Supporting Data S2. Output data of the motif analysis of TTF-1 binding regions in H441 cells using DREME, supporting data for Figure 2F**

500 bp sequences franking the peak summit position of TTF-1 binding regions in H441 cells were used for motif analysis by DREME.

**Supporting Data S3. Output data of the motif analysis of TTF-1-ASCL1 co-binding regions in H209 cells using DREME, supporting data for Figure S3C**

500 bp sequences franking the peak summit position of TTF-1 in the co-binding regions of TTF-1 and ASCL1 in H209 cells were used for motif analysis by DREME.
