# Supplementary material for: Comparative analysis of TTF‐1 binding DNA regions in small‐cell lung cancer and non‐small‐cell lung cancer
Source: Mol Oncol. 2019 Dec 15;14(2):277–93. doi: 10.1002/1878-0261.12608 (PMC6998394; doi:10.1002/1878-0261.12608)
Supplement: Supplementary file 3 — Data S1. Output data of the motif analysis of TTF‐1 binding regions in H209 cells using DREME, supporting data for Figure 2F. [file MOL2-14-277-s003.pdf]

DREME

Discriminative Regular Expression Motif Elicitation

For further information on how to interpret these results please access http://meme-suite.org/.

To get a copy of the MEME software please access http://meme-suite.org.

If you use DREME in your research please cite the following paper:
Timothy L. Bailey, "DREME: Motif discovery in transcription factor ChIP-seq data", *Bioinformatics*, **27**(12):1653-1659, 2011. [full text]

Discovered Motifs | Inputs & Settings | Program Information | Results in Text Format ? NEW | Results in XML Format ? NEW

Discovered Motifs

Next Top

	Motif ?	Logo ?	RC Logo ?	E-value ?	Unerased E-value ?	More ?	Submit/Do ?
1.	RTGASTMA	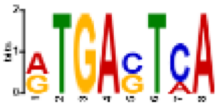	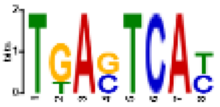	3.5e-2198	3.5e-2198	↧	⇢
2.	RAGTGB	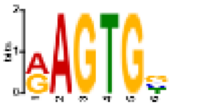	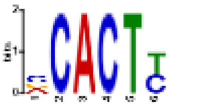	6.3e-864	2.6e-883	↧	⇢
3.	HTTCCB	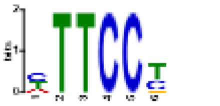	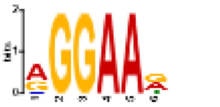	1.0e-399	1.2e-486	↧	⇢
4.	GYAAAYA	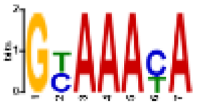	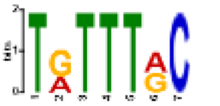	8.7e-373	1.5e-459	↧	⇢
5.	HGTGGTTW	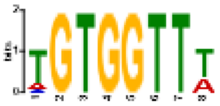	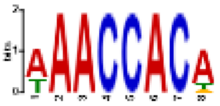	1.7e-265	2.3e-392	↧	⇢
6.	TGARTMA	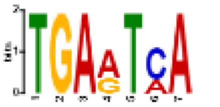	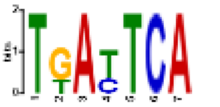	3.6e-238	6.4e-1972	↧	⇢
7.	TYAAGTR	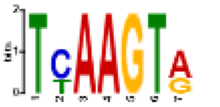	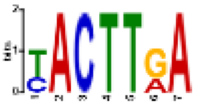	1.2e-202	4.6e-642	↧	⇢
8.	CYCCDCCC	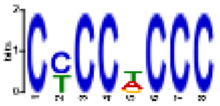	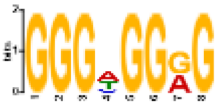	1.2e-175	1.4e-214	↧	⇢
9.	CTCTYVA	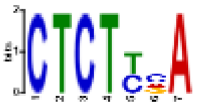	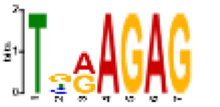	1.4e-162	7.0e-251	↧	⇢
10.	AARTAY	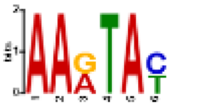	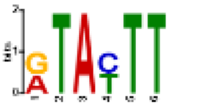	4.5e-093	1.8e-287	↧	⇢
11.	GGAATKY	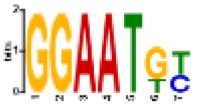	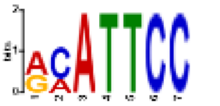	8.8e-090	1.0e-224	↧	⇢
12.	CACGY	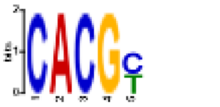	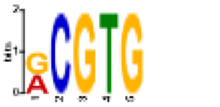	1.4e-087	2.6e-126	↧	⇢

	Motif	Logo	RC Logo	E-value	Unerased E-value	More	Submit/Do
13.	RRCCACA	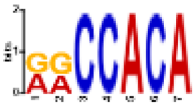	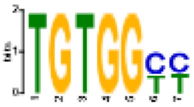	8.7e-074	4.0e-259	↧	⇢

Inputs & Settings

Previous Next Top

Sequences

Source	Alphabet	Sequence Count
./seqs-centered	DNA	51454

Control Sequences

Source	Sequence Count
./seqs-shuffled	51454

Background

Name	Bg.				Bg.	Name
Adenine	0.255	A	~	T	0.255	Thymine
Cytosine	0.245	C	~	G	0.245	Guanine

Other Settings

**Strand Handling**	Both the given and reverse complement strands are processed
**# REs to Generalize**	100
**Shuffle Seed**	1
**E-value Threshold**	0.05
**Max Motif Count**	No maximum motif count.
**Max Run Time**	5217 seconds.

Previous Top

DREME version
5.0.5 (Release date: Mon Mar 18 20:12:19 2019 -0700)

Reference
Timothy L. Bailey, "DREME: Motif discovery in transcription factor ChIP-seq data", *Bioinformatics*, **27**(12):1653-1659, 2011. [full text]

Command line

```
dreme -verbosity 1 -oc dreme_out -png -dna -p ./seqs-centered -n ./seqs-shuffled -t 5217 -e 0.05
```
