# Supplementary material for: Comparative analysis of TTF‐1 binding DNA regions in small‐cell lung cancer and non‐small‐cell lung cancer
Source: Mol Oncol. 2019 Dec 15;14(2):277–93. doi: 10.1002/1878-0261.12608 (PMC6998394; doi:10.1002/1878-0261.12608)
Supplement: Supplementary file 4 — Data S2. Output data of the motif analysis of TTF‐1 binding regions in H441 cells using DREME, supporting data for Figure 2F. [file MOL2-14-277-s004.pdf]

DREME

Discriminative Regular Expression Motif Elicitation

For further information on how to interpret these results please access http://meme-suite.org/.
To get a copy of the MEME software please access http://meme-suite.org.

If you use DREME in your research please cite the following paper:
Timothy L. Bailey, "DREME: Motif discovery in transcription factor ChIP-seq data", *Bioinformatics*, **27**(12):1653-1659, 2011. [full text]

Discovered Motifs | Inputs & Settings | Program Information | Results in Text Format ? NEW | Results in XML Format ? NEW

Discovered Motifs

Next Top

	Motif ?	Logo ?	RC Logo ?	E-value ?	Unerased E-value ?	More ?	Submit/Dow ?
1.	RYAAAYA	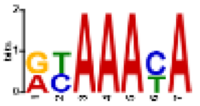	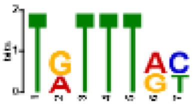	4.5e-511	4.5e-511	↧	⇢
2.	GAGWGB	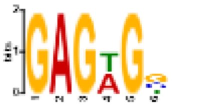	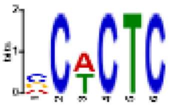	6.2e-396	7.8e-431	↧	⇢
3.	CASSTGB	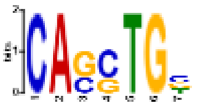	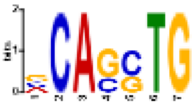	4.3e-249	3.5e-264	↧	⇢
4.	CYCCDCCC	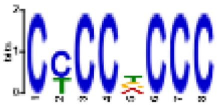	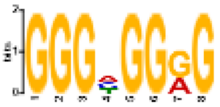	1.0e-128	2.3e-152	↧	⇢
5.	CMGGAAR	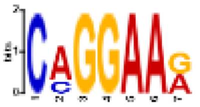	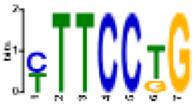	4.2e-102	1.2e-112	↧	⇢
6.	CWCTTVA	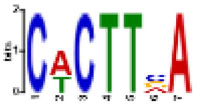	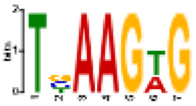	2.2e-095	2.3e-129	↧	⇢
7.	AYAAWRG	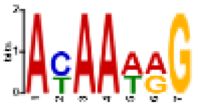	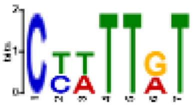	1.3e-102	2.1e-125	↧	⇢
8.	AAAYR	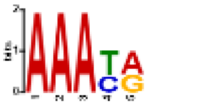	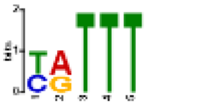	8.6e-053	7.4e-218	↧	⇢
9.	TGARTR	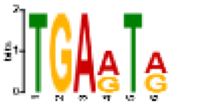	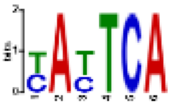	1.4e-040	7.5e-204	↧	⇢
10.	AYAAWGA	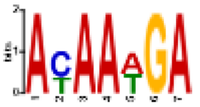	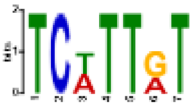	5.4e-029	1.0e-057	↧	⇢
11.	SCACTTA	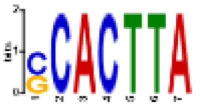	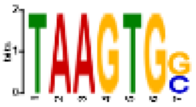	1.4e-025	1.2e-056	↧	⇢

	Motif	Logo	RC Logo	E-value	Unerased E-value	More	Submit/Dow
**12.**	SCHGGGA	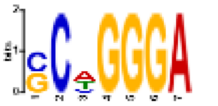	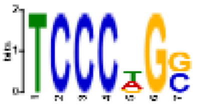	5.5e-024	7.0e-039		
**13.**	CCCGCCYY	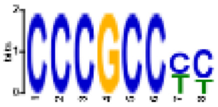	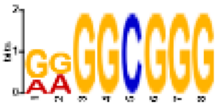	1.2e-021	1.4e-076		
**14.**	TGACRTCA	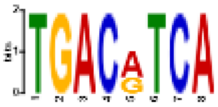	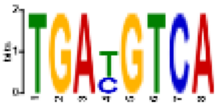	2.0e-020	4.7e-022		
**15.**	AAKTAC	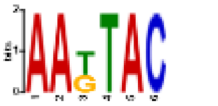	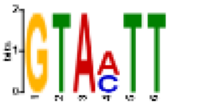	2.2e-019	1.3e-032		
**16.**	AGAGARAG	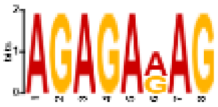	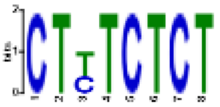	3.6e-014	1.2e-016		
**17.**	TGTTGACW	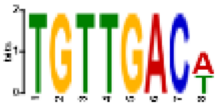	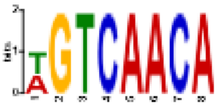	8.3e-012	5.7e-026		
**18.**	CCATCTGY	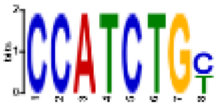	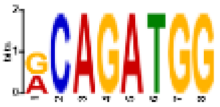	1.1e-011	1.6e-018		
**19.**	CAGGTTWC	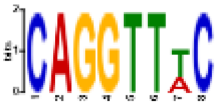	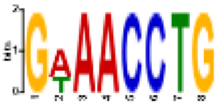	2.6e-011	2.0e-013		
**20.**	GTGGTTW	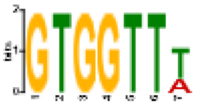	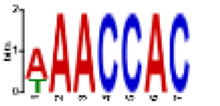	1.1e-010	1.1e-049		
**21.**	CCAATCRG	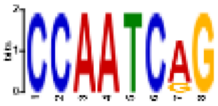	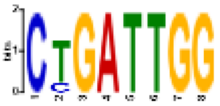	1.2e-009	3.2e-010		
**22.**	AYACACAC	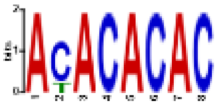	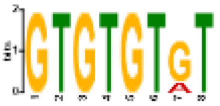	4.0e-009	1.9e-021		
**23.**	CCWCYTCC	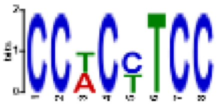	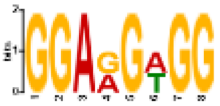	3.9e-011	1.9e-039		
**24.**	TAATKA	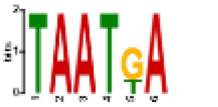	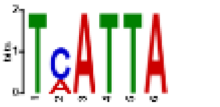	4.8e-009	5.3e-046		
**25.**	CTYCCKCC	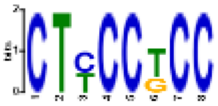	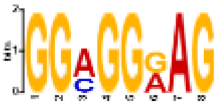	6.3e-009	9.4e-027		
**26.**	ACGTSAC	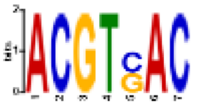	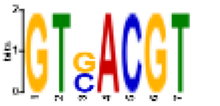	1.7e-008	4.6e-028		
**27.**	AGAKGGCG	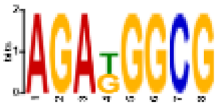	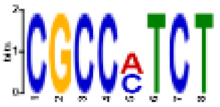	3.2e-008	3.2e-016		
**28.**	CTGGRRA	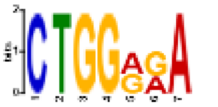	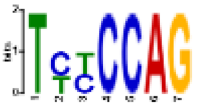	3.4e-009	9.0e-061		

	Motif	Logo	RC Logo	E-value	Unerased E-value	More	Submit/Down
**29.**	CAYRTTAC	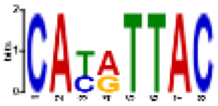	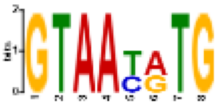	6.8e-009	7.6e-011		
**30.**	GCGCRYGC	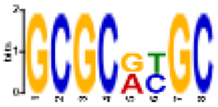	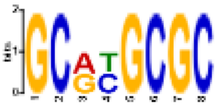	5.7e-008	4.5e-009		
**31.**	AAGTGCYT	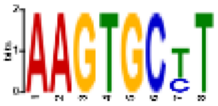	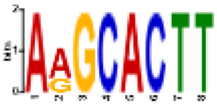	2.2e-007	3.2e-034		
**32.**	TGACTCAB	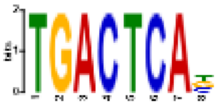	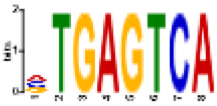	1.3e-006	1.5e-012		
**33.**	CACCGCS	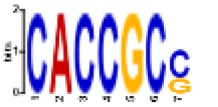	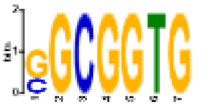	2.6e-006	9.2e-012		
**34.**	CCACRCCC	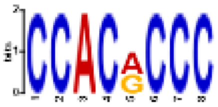	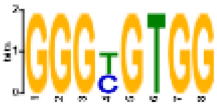	9.5e-006	3.4e-011		
**35.**	AAGWGGAA	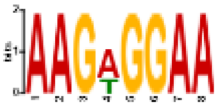	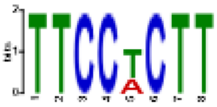	1.5e-005	1.1e-022		
**36.**	ACAAWGCC	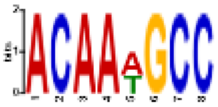	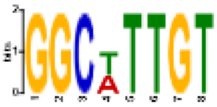	1.9e-005	8.8e-014		
**37.**	AATAAA	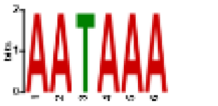	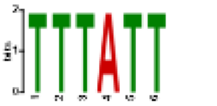	6.2e-005	2.4e-112		
**38.**	TGTACACA	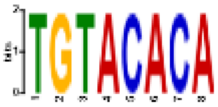	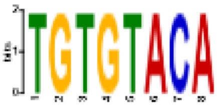	1.2e-004	1.9e-009		
**39.**	GAGDAC	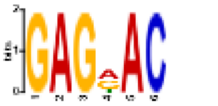	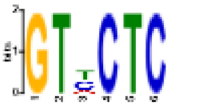	2.0e-004	8.5e-042		
**40.**	CGTCATCA	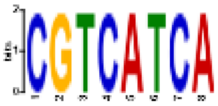	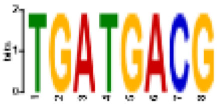	4.6e-004	1.1e-006		
**41.**	AGAGAR	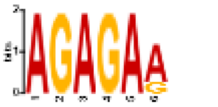	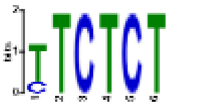	1.0e-003	6.4e-053		
**42.**	GCMATTA	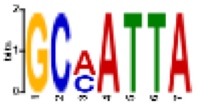	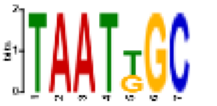	1.8e-003	1.0e-014		
**43.**	CAGCCYGG	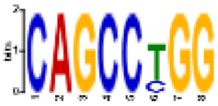	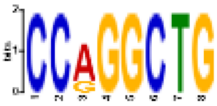	2.5e-003	8.5e-006		
**44.**	GGAAA	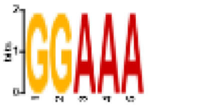	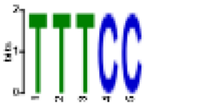	5.0e-003	1.3e-105		
**45.**	ATRGCAAC	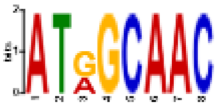	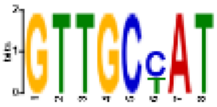	2.5e-002	1.2e-003		

	Motif	Logo	RC Logo	E-value	Unerased E-value	More	Submit/Dowı
46.	AATGARA	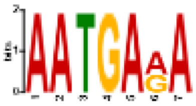	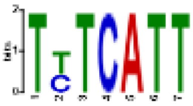	2.8e-002	3.4e-039	↧	⇢
47.	ACAAGWGG	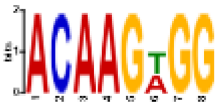	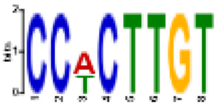	3.8e-002	8.8e-007	↧	⇢

INPUTS & SETTINGS

Previous Next Top

Sequences

Source	Alphabet	Sequence Count
./seqs-centered	DNA	21871

Control Sequences

Source	Sequence Count
./seqs-shuffled	21871

Background

Name	Bg.				Bg.	Name
Adenine	0.263	A	~	T	0.264	Thymine
Cytosine	0.237	C	~	G	0.236	Guanine

Other Settings

**Strand Handling**	Both the given and reverse complement strands are processed
**# REs to Generalize**	100
**Shuffle Seed**	1
**E-value Threshold**	0.05
**Max Motif Count**	No maximum motif count.
**Max Run Time**	5545 seconds.

Previous Top

DREME version
5.0.5 (Release date: Mon Mar 18 20:12:19 2019 -0700)

Reference
Timothy L. Bailey, "DREME: Motif discovery in transcription factor ChIP-seq data", *Bioinformatics*, **27**(12):1653-1659, 2011. [full text]

Command line

```
dreme -verbosity 1 -oc dreme_out -png -dna -p ./seqs-centered -n ./seqs-shuffled -t 5545 -e 0.05
```
