# Supplementary material for: Comparative analysis of TTF‐1 binding DNA regions in small‐cell lung cancer and non‐small‐cell lung cancer
Source: Mol Oncol. 2019 Dec 15;14(2):277–93. doi: 10.1002/1878-0261.12608 (PMC6998394; doi:10.1002/1878-0261.12608)
Supplement: Supplementary file 5 — Data S3. Output data of the motif analysis of TTF‐1‐ASCL1 co‐binding regions in H209 cells using DREME, supporting data for Figure S3C. [file MOL2-14-277-s005.pdf]

DREME

Discriminative Regular Expression Motif Elicitation

For further information on how to interpret these results please access http://meme-suite.org/.
To get a copy of the MEME software please access http://meme-suite.org.

If you use DREME in your research please cite the following paper:
Timothy L. Bailey, "DREME: Motif discovery in transcription factor ChIP-seq data", *Bioinformatics*, **27**(12):1653-1659, 2011. [full text]

Discovered Motifs | Inputs & Settings | Program Information | Results in Text Format ? NEW | Results in XML Format ? NEW

Discovered Motifs

Next Top

	Motif ?	Logo ?	RC Logo ?	E-value ?	Unerased E-value ?	More ?	Submit/Dowr ?
1.	CASCTGB	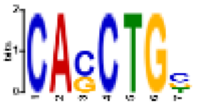	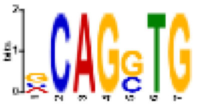	6.2e-433	6.2e-433	↧	⇢
2.	TRTTTR	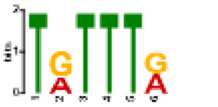	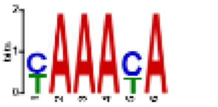	7.1e-119	9.2e-129	↧	⇢
3.	CWCTYM	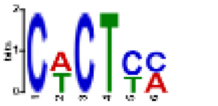	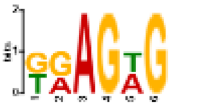	8.2e-053	6.0e-064	↧	⇢
4.	CYCCDCCC	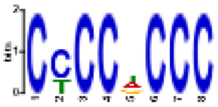	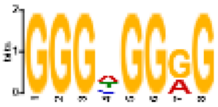	3.0e-033	5.4e-037	↧	⇢
5.	GGAAA	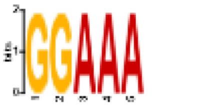	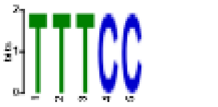	2.0e-030	2.2e-034	↧	⇢
6.	ATAAWR	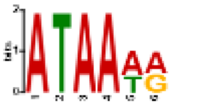	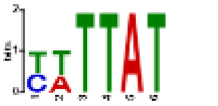	2.4e-021	2.1e-029	↧	⇢
7.	ACAAWRG	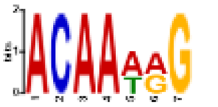	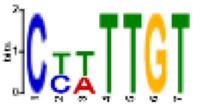	5.6e-014	3.1e-024	↧	⇢
8.	GTAMTY	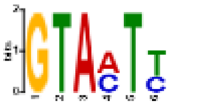	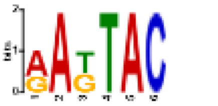	6.6e-013	1.4e-016	↧	⇢
9.	CCCCRGGG	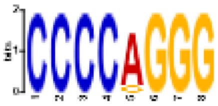	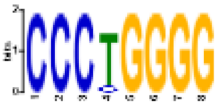	4.0e-012	2.6e-014	↧	⇢
10.	CCATCTGB	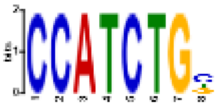	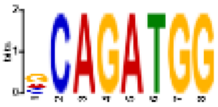	5.6e-009	8.7e-012	↧	⇢
11.	CATTYA	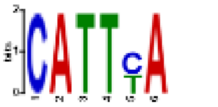	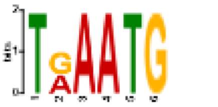	2.6e-008	2.4e-012	↧	⇢

Motif		Logo	RC Logo	E-value	Unerased E-value	More	Submit/Dowı
**12.**	GRGAGR	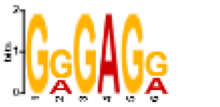	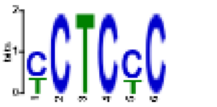	2.2e-008	4.6e-033		
**13.**	RTGASTCA	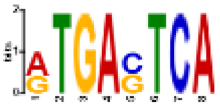	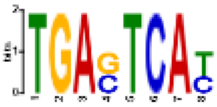	2.5e-007	1.0e-010		
**14.**	CACBTGA	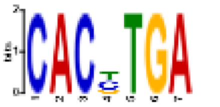	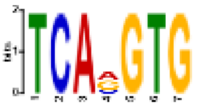	1.7e-007	3.0e-010		
**15.**	CVGGAA	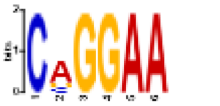	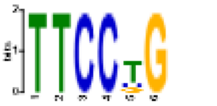	3.8e-005	7.2e-030		
**16.**	ACAAAGM	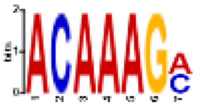	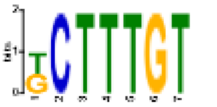	8.4e-004	2.9e-008		
**17.**	DTACACA	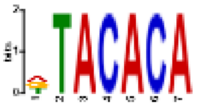	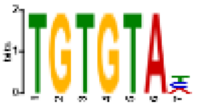	1.2e-003	1.8e-017		
**18.**	GAAAMC	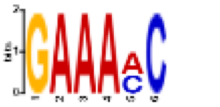	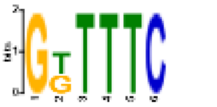	2.0e-003	4.8e-013		
**19.**	CCGTGW	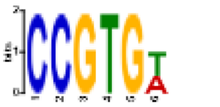	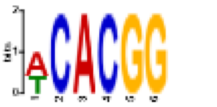	4.9e-003	1.4e-006		
**20.**	ACACACAS	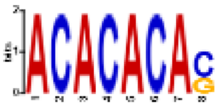	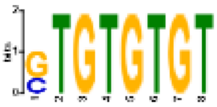	3.3e-002	8.9e-009		
**21.**	CCTGRCAC	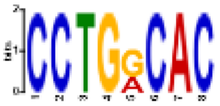	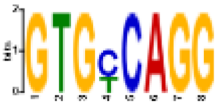	1.4e-002	1.7e-008		
**22.**	CACSTG	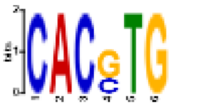	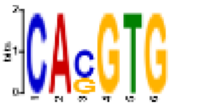	4.5e-002	4.1e-282		

Inputs & Settings

Previous Next Top

Sequences

Source	Alphabet	Sequence Count
./seqs-centered	DNA	5920

Control Sequences

Source	Sequence Count
./seqs-shuffled	5920

Background

Name	Bg.				Bg.	Name
Adenine	0.258	A	~	T	0.259	Thymine
Cytosine	0.241	C	~	G	0.242	Guanine

Other Settings

**Strand Handling**	Both the given and reverse complement strands are processed
**# REs to Generalize**	100
**Shuffle Seed**	1
**E-value Threshold**	0.05
**Max Motif Count**	No maximum motif count.
**Max Run Time**	5304 seconds.

Previous Top

DREME version
5.0.5 (Release date: Mon Mar 18 20:12:19 2019 -0700)

Reference
Timothy L. Bailey, "DREME: Motif discovery in transcription factor ChIP-seq data", *Bioinformatics*, **27**(12):1653-1659, 2011. [full text]

Command line

```
dreme -verbosity 1 -oc dreme_out -png -dna -p ./seqs-centered -n ./seqs-shuffled -t 5304 -e 0.05
```
